# Golden and Silver–Golden Chitosan Hydrogels and Fabrics Modified with Golden Chitosan Hydrogels

**DOI:** 10.3390/ijms23105406

**Published:** 2022-05-12

**Authors:** Marek Kozicki, Aleksandra Pawlaczyk, Aleksandra Adamska, Małgorzata Iwona Szynkowska-Jóźwik, Elżbieta Sąsiadek-Andrzejczak

**Affiliations:** 1 Department of Mechanical Engineering, Informatics and Chemistry of Polymer Materials, Faculty of Material Technologies and Textile Design, Lodz University of Technology, Zeromskiego 116, 90-543 Lodz, Poland; elzbieta.sasiadek@p.lodz.pl; 2 Institute of General and Ecological Chemistry, Faculty of Chemistry, Lodz University of Technology, Zeromskiego 114, 90-543 Lodz, Poland; aleksandra.pawlaczyk@p.lodz.pl (A.P.); adamska.grafik@gmail.com (A.A.); malgorzata.szynkowska@p.lodz.pl (M.I.S.-J.)

**Keywords:** chitosan–Au hydrogel, chitosan–Au microgel, chitosan–Au–Ag hydrogels, chitosan–Au modified fabrics, ICP-QMS, FAAS

## Abstract

Golden and silver–golden chitosan hydrogels and hydrogel-modified textiles of potential biomedical applications are investigated in this work. The hydrogels are formed by reactions of chitosan with HAuCl_4_·xH_2_O. For above the critical concentration of chitosan (c*), chitosan–Au hydrogels were prepared. For chitosan concentrations lower than c*, chitosan–Au nano- and microgels were formed. To characterise chitosan–Au structures, sol–gel analysis, UV–Vis spectrophotometry and dynamic light scattering were performed. Au concentration in the hydrogels was determined by the flame atomic absorption spectrophotometry. Colloidal chitosan–Au solutions were used for the modification of fabrics. The Au content in the modified fabrics was quantified by inductively coupled plasma mass spectrometry technique. Scanning electron microscopy with energy dispersion X-ray spectrometer was used to analyse the samples. Reflectance spectrophotometry was applied to examine the colour of the fabrics. The formation of chitosan–Au–Ag hydrogels by the competitive reaction of Au and Ag ions with the chitosan macromolecules is reported.

## 1. Introduction

Hydrogel materials have been in high demand for over sixty years [1]. They are understood as networks of hydrophilic polymers capable of retaining water in their structure. The specificity of the polymer network and a number of crosslinks in the chain determine the swelling characteristics of hydrogels. Hydrogels can be of a physical or chemical nature with respective chemical interactions, examples of the former being ionic or hydrogen bonds, with the latter being covalent bonds [2,3,4,5]. The nature of hydrogels, consisting of both natural and synthetic hydrophilic polymers, has made them very attractive materials for tissue engineering, cells seeding, wound dressings and drug delivery materials for medical and pharmaceutical applications [6,7,8]. They are also used for other unique applications, such as those related to the manufacturing of complex 3D ionizing radiation dosimetry systems for quality assurance (QA) in radiotherapy dosimetry [9,10,11] and 3D UV radiation measurements [12] or modification of textile materials to give them antimicrobial properties [13].

In this work, the natural polymer of choice to make hydrogels is chitosan [14]. The presence of hydroxyl, amine and acetamide groups in chitosan allows for a number of reactions to be carried out using this polymer. Regarding chitosan hydrogels, they can be formed by crosslinking the cationic chains of this polymer with a number of reactants: β-glycerophosphate; genipin; anionic polymers such as alginate, acrylic acid and *N,N′*-methylenebisacrylamide; glutaraldehyde; β-glycerol phosphate and hydroxyethyl cellulose; pentasodium tripolyphosphate [6,15,16,17,18,19,20]. However, this work is related to the use of noble metals for the preparation of chitosan hydrogels, which also leads to metal nanoparticle formation in the hydrogel structure. It should be mentioned that separate studies have been performed on the preparation and application of noble metal nanoparticles. As such, silver and gold nanoparticles received immense attention. Different methods were presented for their preparation, including sonochemical and chemical reduction, by using ionising radiation or photochemical processes [21,22,23,24,25,26,27,28]. Moreover, there is a broad range of applications of noble metals, for instance, in biotechnology, medicine, electronics and optics [29,30,31,32,33].

It is also important to mention that the combination of gold nanoparticles and chitosan hydrogels are possible to obtain. For instance, the formation of chitosan hydrogels in the presence or absence of acrylic acid and chloroauric acid at a fixed chitosan concentration and elevated temperature has been shown elsewhere [34]. The loading of chitosan hydrogels with noble metals has also been reported [20,35,36]. In other study, gold nanoparticles in chitosan hydrogel were prepared and they were shown to be non-toxic against epidermal cells [37]. In another report, gold nanoparticles (AuNP) in chitosan hydrogel turned out to increase its biocompatibility and it did not show cytotoxicity against mouse embryonic fibroblasts [38]. Additionally, chitosan-based films containing nAu were investigated as materials of high antibacterial activity, showing no cytotoxicity for A549 or for HaCaT cells [39,40]. In summary, chitosan itself can be used for the preparation of hydrogels without noble metals, or such hydrogels can be doped with noble metal nanoparticles to boost their biocompatibility and antimicrobial properties in various biological applications. However, the current study employs only two components of chitosan and gold ions or three components of chitosan, gold and silver ions, and their reaction leads to the formation of golden or silver–golden chitosan hydrogels.

We believe that the formation of chitosan hydrogel structures containing noble metals is strongly dependent on the reaction conditions in which the concentration of the reactants plays an important role [13]. In that work, the macro-, micro- and nanostructures of chitosan–Ag were developed using only two reagents: aqueous solutions of chitosan and silver nitrate. It was discussed that the critical parameter of this reaction is the critical concentration (c*) of chitosan. It was shown that much above c*, the reaction of chitosan with silver nitrate leads to continuous (so-called form from wall-to-wall) transparent hydrogels of amber colour with silver nanoparticles embedded in their structure serving as the cross-linking bonds of chitosan chains (see Figure 1 in [13]). However, below c*, the same reaction leads to the formation of chitosan–Ag micro- and nano-gels (see Table 4 in [13]). This approach associated with the formation of chitosan hydrogels with the use of silver cations as cross-linking agents has been propagated in the research on the reaction of chitosan with hydrogen tetrachloroaurate-(III) hydrate (HAuCl_4_·xH_2_O) presented in the current work. However, the current study is focused more on the investigation of different textile modifications with colloidal chitosan–Au solutions, with respect to the former study. It should be mentioned that various methods of textile/fibre modifications are known, such as: (i) doping the fibres with selected compounds/particles, which can be carried out by mixing the fibre-forming polymer solution with the dopant before preparing the fiber [41], (ii) modification of the textile surface by screen-printing using a printing paste [42,43] or digital printing [44], where the upper part of the textile is modified and the functionality does not exist or is present at much lower concentration on the underside of the textile, and (iii) volumetric modification of the textiles by padding–squeezing–drying, where the entire volume of the textile is uniformly modified by a functionality [13]. In the present work, the last of the methods mentioned was used to modify textiles.

The aims of this work are as follows: (i) to investigate the manufacturing and main features of chitosan–Au hydrogels from nano- to macro-sized structures; for this purpose sol–gel analysis, Vis spectrophotometry and dynamic light scattering were employed, (ii) to use the knowledge gained on chitosan–Au and chitosan–Ag hydrogels and propose the manufacturing of complex chitosan hydrogels containing both Ag and Au in one structure; this was to verify the hypothesis about the possibility of the formation of such hydrogels using various metals thanks to which hydrogel systems with different properties can be obtained, (iii) to use the developed chitosan–Au hydrogels for the modification of different fabrics; for this purpose, the padding–squeezing–drying method was used for the volumetric modification of the textiles, (iv) to examine Au uptake and retention in textiles and hydrogels, and both Au and Ag in hydrogels; this was accomplished with the aid of scanning electron microscopy with an energy dispersive X-ray spectrometer, atomic absorption spectrometry and quadrupole-based inductively coupled plasma mass spectrometry, and (v) to examine both the morphology and the colour of various textiles after modification with colloidal solutions based on chitosan–Au hydrogels (using scanning electron microscopy and reflectance of light measurements); in this case, the optimal effects would be no significant change in the morphology of the textiles and no significant change in the colour of the textiles or no any change in the colour of the textiles after the modification. This paper also suggests the use of both modified textiles and hydrogels as potentially medical or biologically active products. However, such applications have not been investigated in the current work.

## 2. Results and Discussion

### 2.1. Sol–Gel Analysis: Macro-Gels

**Figure 1:**
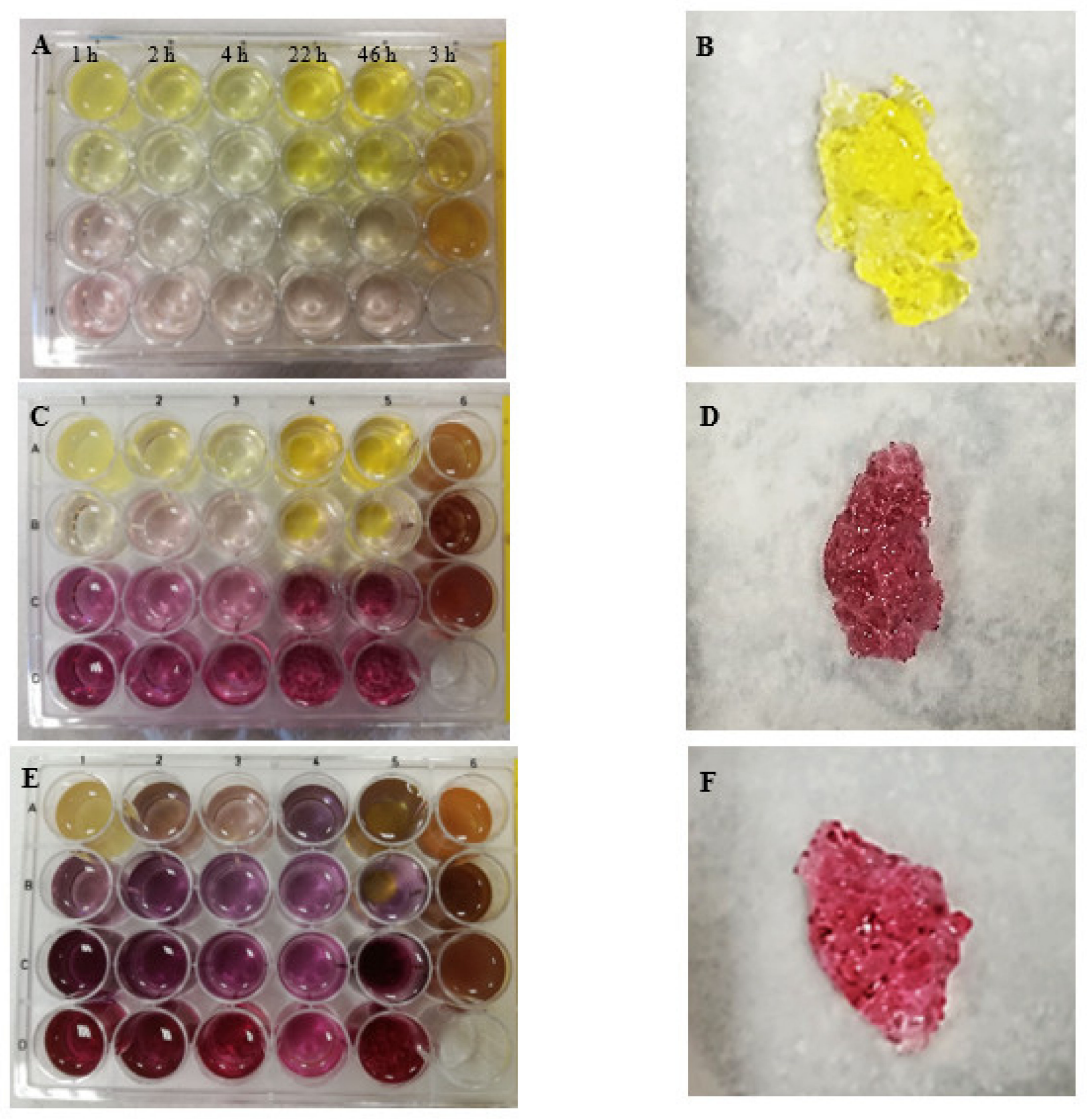
View of the chitosan–Au hydrogel samples. Figure (**A**) is a view of all hydrogel samples prepared in template wells for cell cultivation; the photo was taken approximately 1 h after sample preparation. Figure (**C**,**E**) are the corresponding photographs of Figure (**A**) taken 24 and 48 h after sample preparation. Figure (**B**,**D**,**F**) are sample photographs of chitosan–Au hydrogels prepared from 2% chitosan and 1, 0.1 or 0.05% HAuCl_4_, respectively (reaction time was 46 h). In the template, (**A**–**D**) correspond the concentration of HAuCl_4_ of 1, 0.5, 0.1 and 0.05%, respectively, for vials from 1–5. For vials from 1–5, the reaction times were 1, 2, 4, 22 and 46 h, respectively. Samples 6B, 6C and 6D correspond to a 3-h reaction between 1.5, 1 and 0.5% chitosan, respectively, and 1% HAuCl_4_. The reaction times are marked at the top of photo (**A**). All chitosan–Au hydrogels were prepared at 23 °C. The yellow colour (some samples in Figure (**A**)) is characteristic of the HAuCl_4_ solution.

Reaction of the chitosan solution with HAuCl_4_ at room temperature leads to the formation of products related to the concentration of the reactants. An immediate effect visible to the naked eye is the appearance of the colour of the reacting solutions as shown in Figure 1 (note that the yellow colour is characteristic of the HAuCl_4_ solution). Colour intensity increases with time after preparation. In all cases, the colour of the samples remains yellow or slightly yellow but transparent right after the start of the reaction. However, only after approximately an hour of reaction that some solutions turn pale pink (1, 2, 3 C–D and 4, 5 D) or darker yellow-brown (6 B–C) (Figure 1A). After a 24-h reaction, these colours clearly increase in intensity towards darker pink and brown, respectively (Figure 1C). In addition, a pale pink colour appears in the other samples (1, 2 and 3 B) and a dark pink colour appears in samples 4, 5 C (Figure 1C), while sample 6 A changes from yellow to brownish. The 48-h reaction time causes the brown colour to remain in the brown samples. The pink colour samples become more intense and turn pink-lilac. In addition, samples 2, 3 A change colour from yellow to light brown; sample 4 A transforms from deep yellow to intense lilac; sample 5 A changes colour from yellow to brown; samples 4 and 5 B convert from yellow to lilac (Figure 1E). The samples described above prove the dynamic nature of the reaction of chitosan and hydrogen tetrachloroaurate-(III) hydrate, which is related to the concentration of the reactants and the reaction time. The resulting products are determined by the conditions of the reaction of chitosan with hydrogen tetrachloroaurate-(III) hydrate. The same applies to the appearance of different colours of the reacting mixtures. The colours are associated with the formation of gold nanoparticles of various sizes. This is discussed in Section 3.2. These products obtained were removed from the wells of the cell cultivation well template for sol–gel, Vis spectrophotometry and DLS analyses. Examples of continuous chitosan–Au hydrogels taken out of the wells are presented in Figure 1B,D,F, while observations for the resulting products are summarised in Table 1.

**Figure 2:**
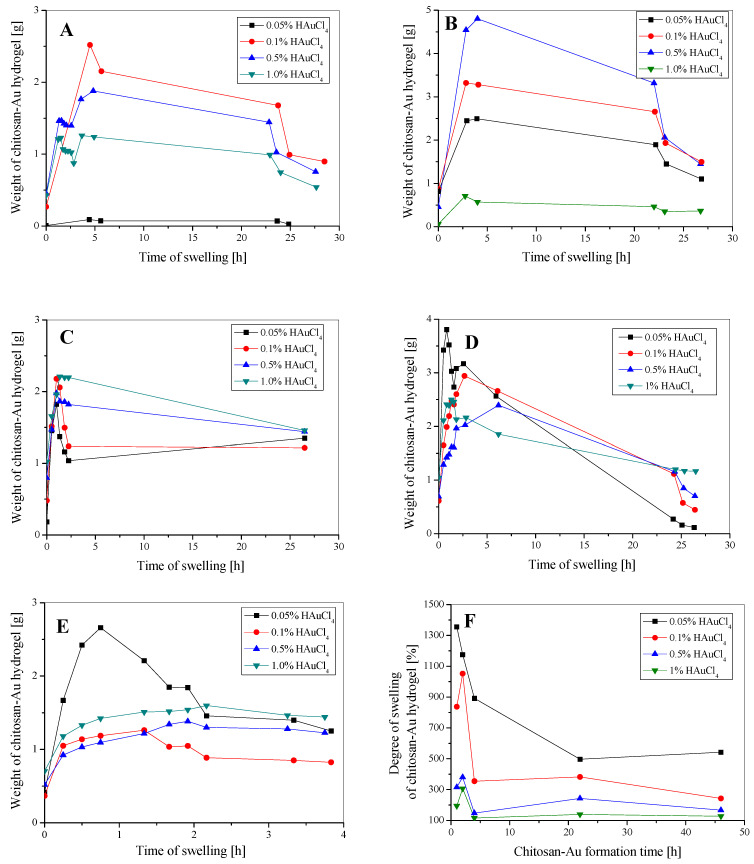
Sol–gel analysis of chitosan–Au hydrogels (**A**–**E**) prepared from 2% chitosan in 1% acetic acid and HAuCl_4_ at the concentrations given in the graphs. The hydrogel formation time is as follows: (**A**)—1 h, (**B**)—2 h, (**C**)—4 h, (**D**)—22 h, (**E**)—46 h. Graph (**F**) corresponds to the degree of swelling of chitosan–Au hydrogels (2% chitosan) with respect to their formation time for the different concentrations of HAuCl_4_ given in the graph.

In Figure 2, the results of sol–gel analysis for chitosan–Au hydrogels are presented; Figure 2A–E corresponds to the weight increase of chitosan–Au hydrogels during swelling for a hydrogel prepared with 2% chitosan and various concentrations of HAuCl_4_, and for the following reaction periods: (A) 1, (B) 2, (C) 4, (D) 22 and (E) 46 h. Figure 2F corresponds to the degrees of swelling of the chitosan–Au hydrogels for the hydrogels made with 2% chitosan and different concentrations of HAuCl_4_. In general, in all examined cases, the immersion of chitosan–Au hydrogels in water resulted in water soaking by the hydrogels increasing their weight. The swelling kinetics are related to both the reaction time of the hydrogel components and their concentrations. Characteristic for all tested hydrogels, with the exception of hydrogels made of 2% chitosan, 1 and 0.5% HAuCl_4_ of 46-h reaction time, is that after a certain period of swelling, they disintegrate. The amount of absorbed water can no longer be retained in the total volume of the hydrogel without maintaining their structural integrity. Low weight chitosan–Au portions are separated from the entire hydrogel structure and are lost during sol–gel gravimetric analysis. In consequence, the characteristic swelling behaviour of hydrogels is that they increase in weight over time, reach their maximum weight and then disintegrate—they lose weight, as illustrated by the points and curves of the results in Figure 2 that grow to peak and then drop down. The disintegration process is typical of such physical hydrogels and is consistent with the observations made for chitosan–Ag hydrogels presented elsewhere [13]. Thus, the swelling properties of physical hydrogels made of a polymer (in this work chitosan) cross-linked with metal ions differ significantly from that of chemical hydrogels having covalent bonds between the chains. The latter may swell in a media until the state of equilibrium, after which they remain integral [4].

It should be noted, however, that the degradation of chitosan–Au hydrogels can be suppressed by using specific conditions for the preparation of these hydrogels. For example, a very long reaction time with a sufficiently high concentration of chitosan and HAuCl_4_ can result in hydrogels with a compact structure, high integrity and greater resistance to degradation. In this way, the gels swell to the maximum degree of swelling and remain integral as observed for the reaction of 2% chitosan with 1 or 0.5% HAuCl_4_ after a 46-h reaction time (Figure 2E). This is consistent with the observations for chitosan–Ag hydrogels [13]. Some hydrogels, such as those made with 5% chitosan and 10% silver nitrate, prepared at longer reaction times (>70 h), were reported to be stable regardless of the pH of the swelling medium and did not disintegrate even when left in water for weeks.

In Figure 2F, the degree of swelling of 2% chitosan–Au hydrogels is reported as a function of reaction time and HAuCl_4_ concentration. It was observed that in all cases, except one for the lowest HAuCl_4_ concentration, the degree of swelling initially increased and then decreased with reaction time. Interestingly, this observation of the initial increase in the degree of swelling is consistent with the observation for chitosan–Ag hydrogels (see Figure 2 in [13]). The decrease in the degree of swelling of chitosan–Au during the reaction is caused by the assumed higher cross-linking density, which prevents the hydrogels from expanding during swelling in water. It should be noted that this observation is consistent with similar behaviour of chemical hydrogels (e.g., [4]) and chitosan–Ag hydrogels [13].

### 2.2. Vis Spectrophotometry and DLS Measurements: Micro- and Nano-Gels

After the removal of the chitosan–Au hydrogels prepared in the 24-well cell culture template, a solution of chitosan reaction products with HAuCl_4_ remains. These products are either derived from unbound substrates by chitosan–Au hydrogels, substrates that have been squeezed out of the shrunken hydrogel, or they are simply nano- and micro-gels and nano- and micro-particles of Au that only appear without the formation of chitosan–Au hydrogels. Undoubtedly, in all these cases, the cationic Au converts into Au particles, changing the colour of the solutions. The Vis absorbance spectra shown in Supplementary Figure S1 confirm the formation of Au particles. It can be seen that the solutions absorb Vis light in the range from 400 to 700 nm. The maxima of bands for the solutions related to A1, B1, D1, A4, B4, C4 and C6 (see Table 2) are given in the graphs in Supplementary Figure S1, and all are located at approximately 550 nm. For samples A4, B1, B4, and C4, the second band can be clearly seen at approximately 600 nm. The spectra indicate the formation of particles with different size distributions. It has also been shown elsewhere that the size of gold nanoparticles affects the position of the peak of the surface plasmon resonance of gold nanoparticles as measured by UV–Vis spectrophotometry [45]. According to this study, for larger nanoparticle diameters, the peak shifts towards higher wavelengths. The DLS analysis confirmed the formation of nano- and micro-structures of chitosan–Au. The DLS results are discussed in Supplementary Materials (Supplementary Table S1).

### 2.3. Flame Atomic Absorption Spectrophotometry (FAAS) of Chitosan–Au Hydrogels

The concentration of gold in each modified hydrogel was quantified after mineralisation in a closed microwave oven system in the reversed aqua regia solution. All the obtained results were expressed in mg/L and related to the decomposition of wet hydrogels for a specific swelling point. This decision was motivated by the extremely low masses of dry hydrogels obtained in this study (which was checked after preliminary studies). Later, the results were calculated with reference to 1 g of chitosan and further discussed. The box-whisker plot (Supplementary Figure S2) for all the collected Au concentration results determined in the modified hydrogel samples showed that most of the observations (75% of the results correspond to the 25% lowest values combined with the 50% most typical) ranged from 35.70 mg/L (lowest values recorded in the hydrogel sample) to 1504 mg/L (value limited by the box length). Although the median, which reached 765.8 mg/L, was placed almost in the centre of the box, it was somewhat different from the mean value (1024 mg/L) (Supplementary Figure S2). The highest result obtained in this study for Au concentration (4586 mg/L) was quantified in a hydrogel sample prepared of 0.5% HAuCl_4_, after 46 h of contact with the reagents and for 15 min swelling time. The highest result obtained in this study for the hydrogels was approximately 120-times higher than the lowest result recorded for the sample of the lowest HAuCl_4_ concentration after 2 h of contact with the reagents and at the swelling point of 250 min (Supplementary Table S2). The initial concentration of HAuCl_4_ seems to have the greatest influence on the results of Au concentration in the hydrogel. For this reason, all applied and examined variants of the hydrogels were then tested for chosen parameters. The influence of each factor was verified by means of statistical tests. The study was further continued with the non-parametric Kruskal–Wallis test employed. The results and discussion are provided in Supplementary Materials (Supplementary Figure S3). The main conclusions after the analyses performed are as follows: (i) for HAuCl_4_ concentration, a predictable increase in Au concentration in hydrogels was observed along with an increase in HAuCl_4_ concentration, (ii) in the case of the influence of contact time, no statistically significant differences were found between the analysed groups, (iii) the increase in the swelling time contributed to the reduction of the residual gold concentration in hydrogels, and (iv) for the influence of the initial chitosan concentration, no statistically significant differences were found in Au levels in the range of the tested chitosan concentrations. The PCA method was further employed to analyse the inter- and intra-individual variation within the studied population of seventy-nine objects with the same initial chitosan concentration of 2% (the remaining variants with a chitosan concentration of 1.5% and 1% were rejected from multivariable analysis due to the low number of representatives (four for each group)). The results are discussed in the Supplementary Materials (Supplementary Figure S4). Four variables were finally selected: the determined Au concentration in the modified hydrogels (range from 35.70 mg/L to 4586 mg/L), initial concentration of HAuCl_4_ (only four options were considered: 0.05%, 0.1%, 0.1% and 1% of HAuCl_4_), swelling time (the lowest point included was 15 min while the highest was 1350 min) and the contact time (1, 2, 4, 22 and 46 h). Briefly, only one variable was negatively correlated with the first and second components; swelling time and contact time, respectively. The amount of Au in chitosan–Au hydrogels and the concentration of HAuCl_4_ were most strongly linked with the first component, whereas contact and swelling time were related to the second component. Au concentration in the hydrogel and HAuCl_4_ concentration are strongly correlated with each other. No correlation was found between the reagents’ contact time during the preparation of chitosan–Au hydrogels and HAuCl_4_ concentration. A negative relationship between the swelling time and the contact time of the reagents suggests that if the contact time between the reagents increases, the swelling of the hydrogels decreases (Supplementary Materials). In general, the longer the reaction time, the more links between chitosan chains are formed, the chitosan–Au hydrogel stiffens and, consequently, the crosslinked chitosan polymer network is less prone to expansion and swelling. This is consistent with the typical behaviour of chemical hydrogels for which a higher degree of crosslinking results in a lower degree of swelling [4].

**Figure 3:**
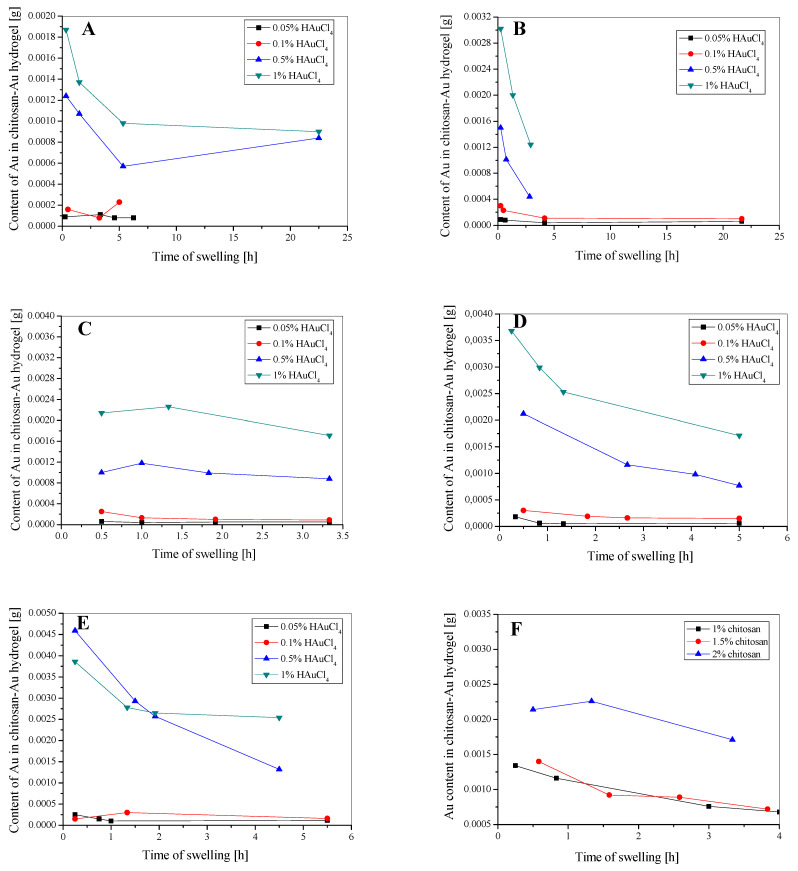
Au content in chitosan–Au hydrogel (1 g) during swelling (sol–gel analysis) in relation to HAuCl_4_ concentration (**A**–**E**) for the hydrogels made of 2% chitosan in 1% acetic acid and for the following formation time: (**A**) 1, (**B**) 2, (**C**) 4, (**D**) 22 and (**E**) 46 h. The graph (**F**) corresponds to the Au content in 1 g of chitosan–Au hydrogel during swelling for the hydrogels made of chitosan with the concentration given in the graph, fixed concentration of HAuCl_4_ of 1% and formation time of 4 h (for 2% chitosan), 3 h (1.5% and 1% chitosan).

The Au content was then recalculated in 1 g of chitosan–Au hydrogels during swelling, which was analysed by the FAAS technique, and the results are shown in Figure 3. Again, the results indicate that Au content is related to chitosan–Au swelling time, HAuCl_4_ and chitosan concentrations. The concentration range recorded for all chitosan-Au hydrogels was 5.0 × 10^−5^–4.6 × 10^−3^ g/1 g of the hydrogel. The highest measured amount of Au equaled to 4.6 × 10^−3^ g/1 g of the hydrogel for the 46-h reaction of 2% chitosan with 0.5% HAuCl_4_ at the beginning of swelling (0.25 h, Figure 3E), while the lowest amount of Au (5.0 × 10^−5^ g/1 g of hydrogel) was measured for a 22-h reaction of 2% chitosan with 0.05% HAuCl_4_ after 5 h of swelling (Figure 3D). The swelling of chitosan–Au hydrogels causes the release of Au from their structure, which reduces the Au content per 1 g of hydrogel. The concentration of chitosan influences the amount of bound Au. No significant differences were observed between the amount of Au bound by chitosan–Au hydrogels made from 1–1.5% chitosan; however, increasing the chitosan concentration to 2% resulted in a much higher Au content per 1 g of hydrogel (Figure 3F).

### 2.4. Modification of Textiles—Reflectance Analysis

Modification of textile materials with chitosan–Au colloidal solutions (undiluted and diluted, Supplementary Figure S5) was carried out using the padding–squeezing method followed by drying at room temperature. This caused a modification of the whole volume of a fabric, contrary to what would be obtained, for example, after screen printing, in which only the surface of textiles would be changed [42]. A number of textiles were selected for this study: polyamide, cotton (bleached and crude), cotton-PET, PET and PAN. The fixation of colloidal chitosan–Au solution is based on both entanglements of the long chitosan chains Au-linked around the fabric macromolecules, and on the chemical interaction between positively charged chitosan chains (as measured in this work) and negatively charged fabrics (negative zeta potential) [46,47]. In consequence, textile and colloid composites were obtained. Their optical properties of the surface were examined by means of reflection spectrophotometry (Supplementary Figure S5). Most of the textiles used in this study either did not change at all or did not significantly change their colour after the deposition of the colloidal solutions irrespective of the concentration of the colloid used. Only crude cotton changed its colour substantially as the reflectance decreased by 34.4, 22.5 and 9.9% at 400, 500 and 700 nm, respectively, for the unmodified and modified samples with the undiluted colloidal solution. In this case, the sample after modification acquired a light pink colour. One can speculate on the reasons behind the colour change of raw cotton. First, raw cotton has a very light brown colour. It contains some impurities on the surface of the fibres, such as proteins, oil and wax, pectin and mineral impurities, which can be a maximum of approximately 12% depending on the type of cotton, i.e., its origin and maturity. Thus, the colloidal chitosan–Au solution may not penetrate deeply into the fibre structure, but remains on the surface, which would also be hydrophobic. The colour of the colloidal solution combines with the natural colour of the raw cotton and reduces the light reflectance as observed in Supplementary Figure S5. However, this problem was not analysed in the present work to assess the penetration depth of the chitosan–Au colloid that would support this assumption. In turn, other fabrics used, including bleached cotton, are free from the mentioned impurities, which most likely changes the resultant colour of the samples after modification and penetration of the chitosan–Au colloid (no significant changes in the reflectance spectra, Supplementary Figure S5).

The reflectance values above 100% from 425–450 nm for cotton-PET, PET and PA denote that a brightening agent was deposited on these fabrics during the manufacturing process of the fabric. In cotton-PET samples, this agent was most likely deposited on PET components. The reflectance results indicate that the bleached cotton fabric modified with the chitosan–Au colloid seems to be less susceptible to colour change than that modified with the chitosan–Ag colloid [13]. In the latter case, a brownish colour could be observed for samples modified with some chitosan–Ag colloids.

### 2.5. SEM-EDS Analysis of the Modified Textiles

**Figure 4:**
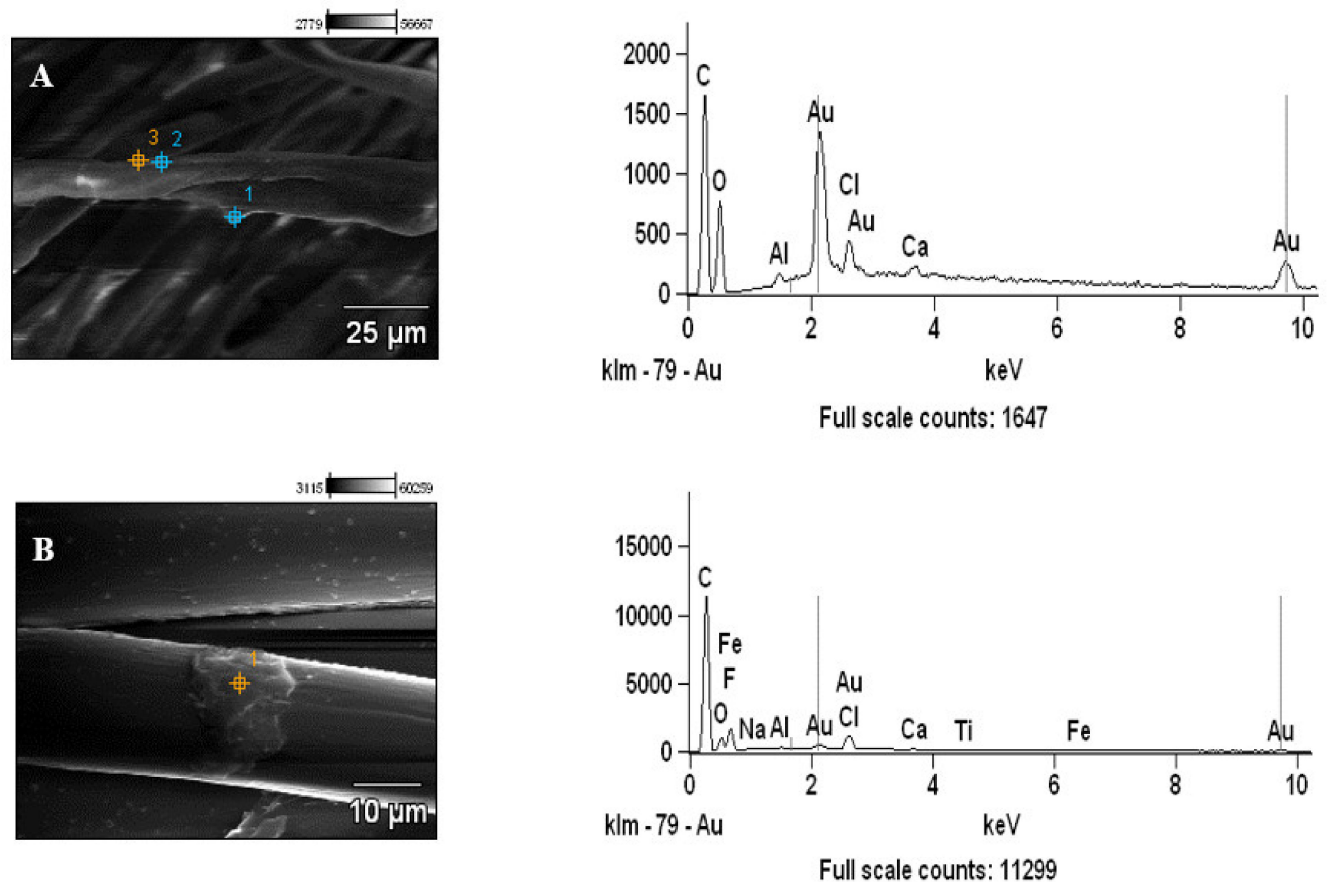
(**A**): SEM micrograph (×1000) with corresponding EDS spectra collected from a single point on the surface of the crude cotton textile. (**B**): SEM micrograph (×2000) with corresponding EDS spectra collected from a single point on the surface of polyester textile.

The morphology (Supplementary Figure S6) and elemental composition (Figure 4) of the textile samples modified with the colloidal chitosan–Au solution were analysed by scanning electron microscopy and X-ray energy-dispersion spectroscopy. Generally, no noticeable changes in the shape of the fibres were observed after the padding–squeezing process was applied. It seems that the obtained SEM images revealed a different behaviour of the chitosan–Au colloid deposited on the fibres. In the case of PET and PA, nano- and micro-size particles can be seen on the fibres. They are more pronounced on PET fibres. These can be Au nanoparticles and dried chitosan–Au colloidal aggregates. In the case of other fibres, a thin film-like structure can be seen covering the fibers without closely forming particles. EDS spectra revealed the peaks which can be attributed to Au presence on the surface of both crude cotton (Figure 4A) and polyester (Figure 4B) fabrics.

All the obtained results were collected for the textiles treated with the original (undiluted) homogenised chitosan–Au hydrogel. The non-flat surface of the tested textile samples severely limited the possibility of performing a confirmatory analysis. Selected areas of a sample were first screened with the YAG detector, which turned out to be helpful in identifying the areas containing heavier atoms compared to the analysed surroundings. The presence of Au was positively verified in the single spot analysis mode. For crude cotton, EDS spectra clearly showed the presence of Au distributed on the fibres; whereas for PET fibres, the obtained results were not as conclusive. For instance, for a selected point suspected to be Au functionality in a PET fibre, EDS spectra showed the presence of Au, which was not confirmed in a further investigation by additional mapping at approximately 30 µm near the region. This, however, should not be surprising after taking into account the overall bulk concentration of Au determined in modified textiles by ICP-MS technique. It was found that with the applied textile modification method and lower concentrations of the colloidal solution (after dilution of the original homogenised hydrogels), Au deposition had to be at a concentration level that was difficult to measure with EDS. The ICP-MS analysis used (next section) confirmed the low concentration of Au deposited on the textiles.

### 2.6. ICP-MS of the Modified Textiles

The statistical analysis of the ICP-MS results (Kruskal–Wallis test) for Au deposited on textile samples by the padding–squeezing–drying was performed. The results obtained are discussed in the Supplementary Materials (Supplementary Figures S7–S9, Supplementary Tables S3–S5). The results of gold content measurements in textile samples modified with homogenised solutions of chitosan–Au hydrogels (the concentration of 0.1% HAuCl_4_) were assessed for two variants of hydrogel formation time: 24 and 48 h, depending on the factors included in this study with nonparametric tests. The following conclusions were drawn for four parameters (contact time between the reagents during the formation of chitosan–Au hydrogels, a specific type of fabric, general type of textile and dilution factor): (i) in the case of the contact time, no statistically significant differences were found between the analysed groups, (ii) natural fibres contained the highest concentration of Au after deposition of the chitosan–Au colloid, (iii) the existence of statistically significant differences between the concentration of gold in the textiles and the general type of textile used was observed, and (iv) a strong correlation between the Au concentration in the textiles and the degree of dilution of the initial chitosan–Au colloid was found (Supplementary Materials).

### 2.7. Chitosan–Au–Ag Hydrogels

We made an attempt to study the formation of chitosan hydrogels through the interaction of chitosan with Au and Ag ions since the possibility of manufacturing chitosan-Ag was previously presented [13]. We were interested in exploring the competition between Au and Ag during hydrogels formation. Thus, the following approach was adopted: (i) chitosan–Au–Ag formation was examined by the reaction of chitosan solution with HAuCl_4_ followed by the addition of AgNO_3_, and (ii) chitosan solution reacted with AgNO_3_ followed by HAuCl_4_ addition. The former was found to serve better than the latter since AgCl precipitated with the latter (when exposed to light, silver was formed that led to the change in colour to dark grey), which was disadvantageous for the hydrogel formation. The results and discussion are presented in the Supplementary Materials (Supplementary Figure S10). In general, the study proved that the formation of chitosan–Au–Ag hydrogels is possible, and that Au preferably links the chitosan macromolecules during the competitive reaction between Au and Ag. This may be due to a higher reduction potential of gold ions than silver ions. This is a preliminary study; however, it points to possible further routes to obtain other types of chitosan hydrogels containing different metals as chitosan macromolecules crosslinkers.

## 3. Materials and Methods

### 3.1. Preparation of Chitosan–Au Hydrogel

Hydrogen tetrachloroaurate-(III) hydrate (99.9% (metals basis), Au 49% min, crystalline, Mw = 339.79 g/mol (anhydrous), HAuCl_4_·xH_2_O, Alfa Aesar, Ward Hill, MA, USA) and chitosan S85/120/A1 (Heppe GmbH, Halle, Germany) were selected for this study. The chitosan was used as delivered by the manufacturer. Its weight-average molecular weight (M_w_), radius of gyration and degree of acetylation (DA) were assessed in a previous study [13] and are equal to 261,500 g/mol, 61.5 nm, 12%, respectively. The streaming current was measured (PCD 03 pH, Mütek, Filderstadt, Germany) for a 0.1% chitosan solution in a 1% acetic acid, which resulted in 1250 mV, indicating that this polymer solution is positively charged.

Chitosan–Au continuous hydrogels were prepared in line with the chitosan–Ag hydrogel preparation procedure proposed elsewhere [13]. According to this method, an aqueous solution of the crosslinker (HAuCl_4_·xH_2_O) is poured over a solution of chitosan in 1% acetic acid (Chempur, Piekary Śląskie, Poland). In this work, 1.5 mL of HAuCl_4_·xH_2_O solution was added on the top of the same volume of chitosan solution in one well of a 24-well plate, and the reagents were left without steering, protected from daylight, for the predetermined time at ~23 °C. The reagent concentration ranges and reaction conditions are given in Table 2. It should be noted that decomposition of chitosan during the reaction with HAuCl_4_·xH_2_O was not observed organoleptically for the reaction carried out at room temperature and the reaction time selected in this study. However, preliminary studies have shown that the same reaction performed at elevated temperatures may be counterproductive for the hydrogel formation and may lead to chitosan degradation and the conversion of chitosan–Au hydrogels to viscous solutions. This, however, requires further investigations and has not been examined in detail in the current study.

**Scheme 1:**
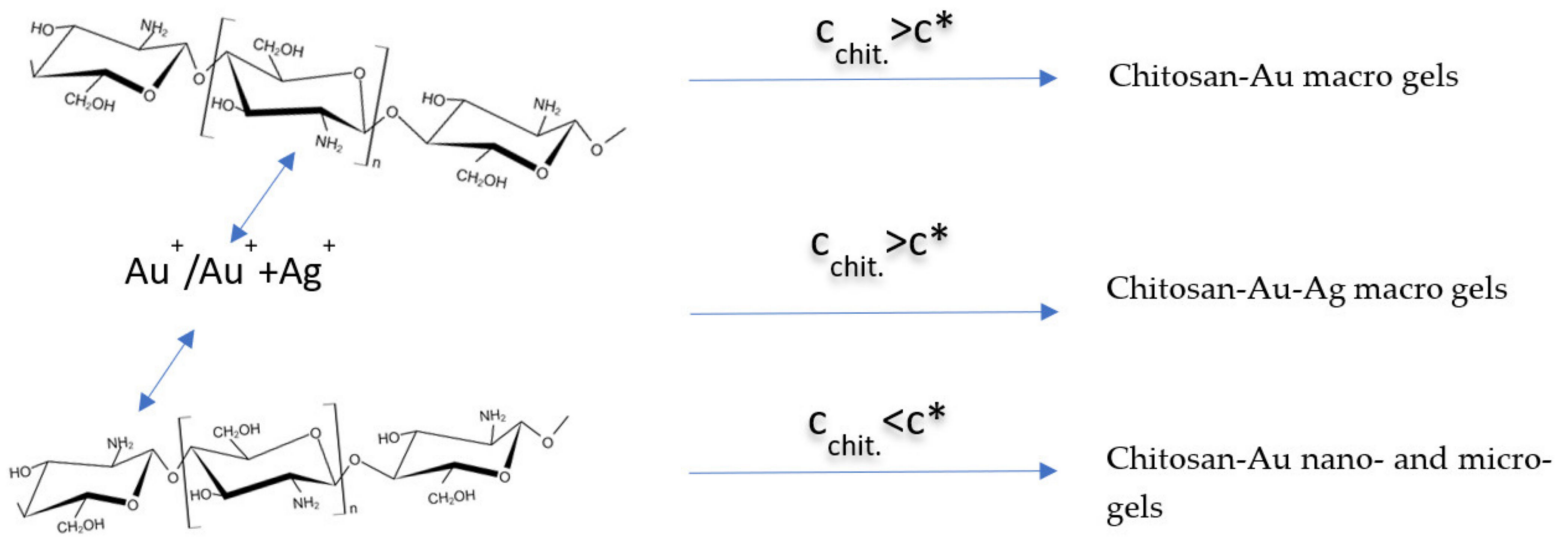
The concept of formation of chitosan–Au nano, micro- and macro-gel structures, and chitosan–Au–Ag macro-gels.

The conditions for the preparation of chitosan–Au hydrogels were chosen based on our former results obtained for chitosan–Ag hydrogels [13] related to the study of the critical concentration of chitosan, c*. The intrinsic viscosity measurements of chitosan in a 1% acetic acid revealed [η] = 2870 mL/g and, thus, c* = 0.035%. This value of c* indicated the transition from dilute to semi-concentrated chitosan solution. If the concentration of chitosan is above c*, its polymer coils should attach and even interpenetrate. Below c*, the coils should be separated. The studies of the chitosan–Ag hydrogel preparation showed that for chitosan concentrations above but close to c*, only more viscous solutions of chitosan–Ag can be formed. In order to form from wall-to-wall hydrogels, so that they can be removed from the preparation vial as the entire structure similar to a continuous hydrogel, the concentration of chitosan must be much higher than c*. Consequently, in the current study, the concentration range of a chitosan solution from 0.5–2% was selected and adopted as appropriate to present both the formation of the continuous chitosan–Au hydrogel at higher chitosan concentrations, and the micro- and nanogels of chitosan–Au at lower chitosan concentrations. Initial experiments were carried out before starting the studies described in this paper in order to verify the concept of chitosan–Au hydrogels formation using chitosan and HAuCl_4_·xH_2_O at selected concentrations and ratios, and the reaction time was considered to be at least one hour to form continuous hydrogels. After preliminary experiments, the set of conditions described in Table 2 was decided. The concept of the experiment regarding the formation of chitosan–Au and chitosan–Au–Ag (Section 2.6) structures is shown in Scheme 1.

### 3.2. Sol-Gel Analysis

Continuous chitosan–Au hydrogels were examined by testing their swelling in distilled water. Samples were immersed in 200 mL of water, removed and weighed at specified intervals. Before the next immersion, the water was changed. The degree of swelling (DS) was calculated using the expression DS [%] = (m_w_/m_gel_) × 100%, where m_w_ denotes the weight of water absorbed by the chitosan–Au hydrogel, and m_gel_ is the weight of the chitosan–Au hydrogel prior to immersion in water. Note that each experimental point corresponds to one hydrogel measured; thus, no experimental errors associate the points in the corresponding graphs of Section 2.

### 3.3. Vis Spectrophotometry Measurements

Vis absorbance spectra were recorded for solutions remaining after the preparation of chitosan–Au gels (A1, B1, D1, A4, B4, C4, Table 2) and for reaction products of chitosan with HAuCl_4_·xH_2_O (C6 in Table 2) using a UV-Vis spectrophotometer (Jasco V-530, 190–1100 nm, Tokyo, Japan). Measurements were performed against a baseline from a cuvette filled with distilled water (which was further subtracted from the absorbance spectrum of the measured sample). Each measurement lasted from 2–3 min, and each sample was measured in the wavelength range from 400–800 nm with a resolution of 1 nm.

### 3.4. Dynamic Light Scattering Measurements

Dynamic light scattering measurements (DLS, Particle Size Analyser, NICOMP, Kristel Circle, Port Richey, USA) were performed for chitosan–Au nano/microgels solutions remaining after the preparation of chitosan–Au gels (2% chitosan): variants A1, B1 and D1 for a 1-h reaction with 1, 0.5 and 0.05% HAuCl_4_, respectively; whereas, A4, B4 and C4 refer to a 22-h reaction with a 1, 0.5 and 0.1% HAuCl_4_, respectively. Sample C6, corresponding to a 0.5% chitosan in a 3-h reaction with a 1% HAuCl_4_, was also measured. Measurements were made at 23 °C. Each sample was measured in duplicate. The accuracy of the DLS instrument is a one decimal place.

### 3.5. Modification of Fabric with Chitosan-Au Hydrogel

A batch of chitosan–Au hydrogels was obtained after the reaction of a 2% chitosan (5 mL) with a 0.1% HAuCl_4_·xH_2_O (5 mL) for 24 or 48 h. The hydrogels were swelled in distilled water to the maximal degree of swelling for 4 h. During the swelling, the distilled water was changed twice (each time after 2 h swelling) to remove unbound HAuCl_4_·xH_2_O. Afterwards, the swollen hydrogels (22 g) were homogenized with 78 mL of distilled water. Homogenisation yielded 100 mL of chitosan–Au colloidal solution. Then, five dilutions of the colloid were prepared in a such a way that the original solution was diluted 2, 4, 8, 16, 32 times. Each solution obtained was used to modify the following textiles: polyester (PET), polyamide (PA), polyacrylonitrile (PAN), cotton (crude and bleached) and cotton-PET (or C/PET). The basic metrological properties of the fabrics are shown in Table 3.

The fabrics were washed beforehand in a washing liquor with a non-ionic detergent (0.5 g/dm^3^ Rokafenol N8-P7, PCC Rokita SA, Brzeg Dolny, Poland). The samples were mixed for 30 min in the liquor from 65–70 °C simulating the washing process, and then rinsed several times with tap water (65–70 °C), cold tap water and distilled water. The washed fabrics were dried at 90 °C for 2 min (Binder, Tuttlingen, Germany). Colloidal solutions of chitosan–Au hydrogels were used for volumetric modification of the fabrics by the padding–squeezing–drying method (clamp 45 kg cm, E. Benz, Rümlang-Zürich, Switzerland). Drying was at 23 °C overnight.

The rationale for the conditions of the experiment described in this section is as follows. For the modification of textiles with the chitosan–Au hydrogel, an exemplary hydrogel was selected for which swelling to the maximum degree of swelling had to be initiated shortly after the required reaction time between the reagents. The swelling process to the maximum degree of swelling had to be relatively short (several hours) for the convenience of the entire experiment, preparation of colloidal hydrogel solutions and the padding–squeezing–drying process in one day. It should be noted that the selection of hydrogels for textile modification is not limited to the example described above; however, the extension of this experiment was beyond this work.

### 3.6. Chitosan–Au–Ag Hydrogels

The conditions for the preparation of chitosan–Au–Ag hydrogels were established after preliminary tests of the preparation of such gels. Note that reagent concentrations, ratios and reaction times are unlikely to be limited to only those outlined in this section. However, extending this experiment was scheduled for other studies. The hydrogels were prepared by analogy to chitosan–Au hydrogels. However, 3 h after the addition of 0.1% HAuCl_4_·xH_2_O (1.5 mL), 0.1% AgNO_3_ (1.5 mL) (Chempur, Piekary Śląskie, Poland) was added to a 2% chitosan solution (3 mL) in 1% acetic acid, and the reaction between the reagents was continued for 24 h. This reaction was repeated for the following conditions: 24 h after the addition of 0.5% HAuCl_4_·xH_2_O (1.5 mL), 0.5% AgNO_3_ (1.5 mL) was added to a 2% chitosan solution (3 mL) in 1% acetic acid and the reaction between the reagents was continued for 48 h. Then, the hydrogels were assessed for Ag and Au content in the dried hydrogels after the swelling process to the maximum degree of swelling. The remining solutions in the reaction wells after the removal of the hydrogels were assessed for the content of Au and Ag as well. The concentration of Au and Ag was analysed by Flame Atomic Absorption Spectrometry (FAAS).

### 3.7. AAS

The selection between spectroscopic instrumental techniques for Au determination was made in reference to the predicted level of Au in the studied material. Due to the expected relatively high levels of Au in the hydrogels, the atomic absorption spectrometry (AAS) technique was chosen. In order to measure Au concentration in chitosan–Au hydrogels (with the exception of chitosan–Au–Ag hydrogels) by the flame atomic absorption spectrophotometer (FAAS, Solaar M6, UNICAM, Budapest, Hungary) with a deuterium (D2) lamp, it was necessary to remove unbound tetrachloroaurate-(III) hydrate. This was performed by immersing the hydrogels in distilled water (swelling medium) and measuring their weight while swelling (sol–gel analysis described above). The hydrogel samples (wet mass of hydrogels for a particular swelling time has not exceeded 4 g) were then mineralised in reverse aqua regia (HNO_3_ to HCl was equal to 3:1 *v/v*) using microwave energy (UltraWave, Milestone, Shelton, CT, USA). Concentrated nitric acid (Baker, Chicago, IL, USA) and concentrated hydrochloric acid (Chempur, Piekary Śląskie, Poland) were analytical grade. The parameters of the mineralisation process of the hydrogel samples were similar to the ones recommended for the decomposition of polymers. The procedure consisted of two steps: during the first 25 min, the maximal microwave power (up to 1500 W) was adjusted in such a way that at the end of this step the temperature inside the reactor reached 240 °C; the second step lasted for 10 min, during which the temperature inside the reactor was sustained at 240 °C. The maximum allowed pressure during this decomposition did not exceed 110 bar. Then, the samples were quantitatively transferred into volumetric flasks and filled up to a volume of 50 mL. Next, the measurements of gold concentrations in the solutions were performed at the wavelength of 242.8 nm. The applied parameters included: bandpass of 0.5 nm; burned height of 11.0 mm; flame: air-acetylene with a fuel flow rate of 0.8 L/min. In our case, the background correction was also employed. The calibration curve was created by the proper dilution of Au (Merck, Darmstadt, Germany) with a nominal concentration of 1000 mg/L, and covered a wide range from 20 mg/L up to 150 mg/L. The established limit of quantification for the FAAS technique was set up at 0.48 mg/L. Due to the lack of the proper certificate reference material with a certified content of Au and similar matrix, the laboratory-made reference materials were prepared. The correctness of the whole applied procedure was positively verified based on the obtained recovery values after doping the sample containing mineralised unmodified cotton fabric with a known content of Au solution, and a satisfactory agreement was achieved between the theoretical value and obtained results.

### 3.8. ICP-QMS

As a second technique of Au level measurement, quadrupole-based inductively coupled plasma mass spectrometry (ICP-QMS) was chosen. Since the level of Au in textiles modified with the highest dilution degree of the primary homogenised chitosan–Au hydrogels was expected to be low, all chitosan–Au modified textile samples were finally examined by inductively coupled plasma mass spectrometry with a quadrupole analyser, ICP-QMS (X-Series, Thermo Electron Corporation, Waltham, MA, USA). The samples (approximately 0.2–0.25 g) were mineralised (UltraWave system, Milestone, Shelton, CT, USA) in reverse aqua regia (HNO_3_: HCl = 3:1 *v/v*) with microwave energy using a program dedicated to the decomposition of cotton fabric. During the first 15 min, the maximal microwave power (up to 1500 W) was adjusted in such a way that at the end of this step the temperature inside the reactor reached 220 °C, the second step lasted for 10 min, during which the temperature inside the reactor was sustained at 220 °C. The maximum allowed pressure during this decomposition did not exceed 100 bar. Next, the samples were quantitatively transferred into volumetric flasks, filled up to the final volume of 50 mL with deionised water. In the case of the polyester-based textiles, there was a need for the additional filtering of samples with the use of the pressure pump. Then, the measurements of Au concentrations in the solutions this way obtained were made. In the case of the samples in which a relatively large amount of retrained Au was suspected, they were additionally diluted from 10 to 1000-times by subsequent dilution just before the measurement (e.g., for textiles modified with the primary homogenised chitosan–Au hydrogel solution). Moreover, this technique was used to assess the Ag content in chitosan–Au–Ag hydrogels. The measurements of gold and/or silver concentrations in the obtained solutions were performed for the following stable isotopes: ^197^Au and ^107^Ag. The applied parameters included: the argon as a plasma gas (auxiliary gas approximately 0.8 L/min, cooling gas 13.2 L/min); concentric quartz nebuliser with a carrier gas flow of 0.88 L/min; quartz torch; plasma power of 1340 W; the detector voltage 3190 V. The calibration curve was created by the proper dilution of a base single-element solution of Au (Merck, Darmstadt, Germany) and a base single-element solution of Ag (Merck, Darmstadt, Germany) and both with a nominal concentration of 1000 mg/L, and covered a wide range from 2 µg/L up to 100 µg/L. The formed chitosan–Au–Ag hydrogels were treated in the same way as hydrogels containing only Au. Again, wet hydrogels for a particular swelling time were decomposed using the reverse aqua regia. Additionally, the residue water after the process of hydrogel swelling was studied in order to assess the degree of Au or Ag bonding with the hydrogel matrix. For this purpose, approximately 5 mL of the sample after swelling was decomposed with 2 mL of reverse aqua regia (1.5 mL of nitric acid and 0.5 mL of hydrochloric acid) and finally diluted up to a volume of 12 mL. The established limit of quantification for the ICP-MS technique was set up at 0.07 µg/L. Due to the lack of the proper certificate reference material with a certified content of Au and similar matrix, the laboratory-made reference materials were prepared. The correctness of the whole applied procedure was positively verified based on the obtained recovery values after doping the sample containing the mineralised unmodified hydrogel with a known content of Au solution, and a satisfactory agreement was achieved between the theoretical value and obtained results.

### 3.9. Statistical and Chemometric Analysis

STATISTICA 10 (New York, NY, USA) software was employed for statistical and multivariate analysis. Due to the significant amount of the quantitative data, the results were presented in the form of a box and whisker plot on an interval scale. These diagrams, with large numbers of observations, can be especially helpful in depicting the general distribution trend and an indication of potential outliers in the data set. As a central value the median was chosen. The box was limited by the 2nd and 3rd quartiles, while the length of whiskers represented the distribution of results within the 1st (lower) and 4th (upper) quartiles extended to the lowest and to the highest observations, respectively. The size (length) of the box is correlated with the variation of results with 50% of the most typical values. Thus, the bigger the box is, the higher variation of the most typical results can be observed. The obtained graphs summarised the generated data sets with regard to studied parameters, for example, the initial concentration of the Au precursor (HAuCl_4_), the swelling point, the contact time between the chitosan and Au precursor or the fabric type, etc.

Before any statistical analysis, the distribution of studied variables (Au concentration) was checked by a normality test such as the Kołmogorov–Smirnov test for the accepted level of significance α = 0.05. When the null hypothesis regarding the normal distribution was rejected, the nonparametric test, the Kruskal–Wallis test, was engaged. This test was helpful in assessing the importance of statistical differences in Au concentration among studied groups, which were formed in order to identify the influence of the chosen criteria. Additionally, principal component analysis (PCA) was performed, where the separate clusters of cases can be potentially distinguished as a graphical illustration in an adaptive way of a wider dataset in the mean of low dimensional representations. In general, in the PCA method new uncorrelated variables are formed, while the variance is successively maximized. In our case, due to a low number of variables, no reduction of data was suggested. This method gave us a chance to trace the factors which were the most likely responsible for the specific cluster formation.

### 3.10. Reflectance Measurements

The reflectance measurements of the modified textile samples were made with the following spectrophotometer: Spectraflash^®^ SF 300 UV^®^, Datacolor, Applied Color Systems Inc., Lawrenceville, NJ, USA. The instrument has been calibrated before the measurements. The 190–400 nm UV light was cut off in order not to expose the samples to UV light. The reflectance of light (R [%]) was measured from 400–700 nm with 10 nm resolution (the resolution available for this device).

### 3.11. Microscopic Analysis

The morphology of the samples was examined using scanning electron microscopy (SEM, TESCAN VEGA3—EasyProbe with Energy Dispersive X-ray spectrometer, Xflash Detector 410-M; Bruker, Billerica, MA, USA). The samples were sputtered with Au/Pd in a Cressington 108 Auto Sputter Coater for adequate contrast. Additionally, the presence on the surface of the gold particles, both in the hydrogels and in the textiles treated with primary homogenised hydrogels, were studied by scanning electron microscope (SEM) HITACHI S-4700 (Tokyo, Japan) equipped with an energy dispersive X-ray spectrometer (EDS) by Thermo NORAN (Waltham, MA, USA). Before measurements, the samples were placed on carbon plasters and coated with carbon in the Cressington 208 HR system (Watford, UK). Due to a relatively low concentration of gold on the surface of the studied samples (typical detection limits for this technique are approximately 0.1 wt.%), the spot analysis was employed. In order to locate the areas rich in gold, first, the YAG detector was used. For this detector in which back scatter electrons (BSE) are applied, the signal decreases with the decreasing mass of analysed element. Thus, heavy masses can be discriminated from the background of much lower masses as the bright spots in the SEM micrographs. After the areas potentially enriched with Au were traced, the confirmatory X-ray microanalysis was performed at a magnification of ×1000. For selected micro-areas, the maps of the distribution of the chosen elements at higher magnifications of ×1000 and ×2000 were also collected.

## 4. Conclusions

This work presents the preparation, characterisation and application of chitosan–Au structures in the form of micro- and macro-hydrogels. The preparation and characterisation of chitosan–Au–Ag macro-hydrogels is also shown.

Most fabrics did not change colour after volumetric modification with chitosan–Au colloids, however, they contained chitosan–Au attached to the fibres. The Au concentration in the fabrics was strongly related to the initial concentration of the chitosan–Au colloid, while the contact time between the reagents in the hydrogel formation step did not appear to play a significant role, and the natural fibres are more susceptible to the attachment of chitosan–Au than synthetic fibres.

The proposed formation of chitosan–Au–Ag hydrogels opens up new possibilities for obtaining complex chitosan hydrogels containing various metals in their structure. It also seems that the chitosan–Au and chitosan–Au–Ag hydrogels can be further investigated for the development of medical products, including wound healing sponges. Additionally, the results obtained indicate the possibility of fabric modification to prepare chitosan- and gold-containing antimicrobial textiles.

## Figures and Tables

**Table 1:** Naked eye observations of chitosan–Au hydrogels during transfer of the gels to the sol–gel analysis beakers. The table corresponds to a 24-well template for cultivation of cells (Figure 1, Table 2).

Concentration of HAuCl_4_	Sample Number						
	1	2	3	4	5	6	
	Reaction Time between 2% Chitosan and HAuCl_4_						
	1 h	2 h	4 h	22 h	46 h	3 h	
						1% HAuCl_4_ + Chitosan	
A 1%	Intense yellow gel Viscous, liquid jelly-like structure that was hard to transfer to a beaker	Yellow gel Firm gel resembling gelatine jelly	Yellow gel Firm gel resembling gelatine jelly	Yellow gel Very firm gel from wall-to-wall	Yellow gel Very firm gel from wall-to-wall	Yellow gel Very firm gel from wall-to-wall that was easy to transfer to a beaker	1.5% chitosan
B 0.5%	Bright yellow gel Gel resembling light gelatine jelly; it was possible to transfer the whole gel to a beaker	Bright yellow gel Firm gel resembling gelatine jelly (from wall-to-wall)	Bright yellow gel Firm gel resembling gelatine jelly	Yellow gel Firm gel resembling gelatine jelly (not from wall-to-wall)	Yellow very firm gel Gel from wall-to-wall. This gel was the stiffest out of the other gels in series 5	Yellow-brownish bright gel Viscous gel that was hard to transfer to a beaker	1% chitosan
C 0.1%	Bright transparent light pink viscous gel	Bright yellow gel Gelatine jelly-like structure	Bright yellow gel Gel resembling light gelatine jelly	Pink-raspberry bright gel In the vial it looked like raspberry strings but kept its shape during swelling	Raspberry-colour gel Viscous gel	No typical gel from wall-to-wall; yellow-brownish solution	0.5% chitosan
D 0.05%	Bright transparent light pink viscous gel No typical gel from wall-to-wall was formed. The mixture remained as a transparent solution	Bright pink gel Viscous gel	Bright pink gel Gel resembling viscous physical gel	Bright pink gel Firm gel that looked in a vial like raspberry-colour strings	Bright raspberry-colour viscous gel	-	

**Table 2:** Conditions for the preparation of chitosan–Au hydrogels, micro- and nanogels; chitosan concentration in a 1% acetic acid was 2% (*w/v*) except for the options from 6 A–C, which corresponds to the reaction of a chitosan solution from 0.5–1.5% with 1% HAuCl_4_. The table reflects the 24-well plate used in the experiment. The preparations were carried out at 23 °C.

Reaction time (h)		1	2	4	22	46	3
HAuCl_4_ (%)	Options	1	2	3	4	5	6
1	A	+	+	+	+	+	1.5% chit
0.5	B	+	+	+	+	+	1% chit
0.1	C	+	+	+	+	+	0.5% chit
0.05	D	+	+	+	+	+	-

**Table 3:** Basic features of the fabrics chosen for the study.

Fabric	Crude Cotton	Bleached Cotton	Cotton/PET	PET	PA	PAN
Weave	plain	plain	twill	plain	plain	twill
Warp [1/dm]	250	280	500	600	400	240
Thread [1/dm]	220	260	240	400	300	180
Thickness [mm]	0.40	0.36	0.36	0.14	0.12	0.57
Surface mass [g/m^2^]	150	140	185	80	65	220
Color	beige with visible seed flakes	white	white	white	white	beige
Optical brightener	no	no	yes	yes	yes	no

## Data Availability

Not applicable.

## Supplementary Materials

The following supporting information can be downloaded at: https://www.mdpi.com/article/10.3390/ijms23105406/s1.

## References

[1] Wichterle O., Lím D. (1960). Hydrophilic gels for biological use. Nature.

[2] Peppas N. (1986). Hydrogels in Medicine and Pharmacy.

[3] Ulanski P., Rosiak J.M. (1999). The use of radiation technique in the synthesis of polymeric nanogels. Nucl. Instrum. Meth. B.

[4] Kozicki M. (2011). How do monomeric components of a polymer gel dosimeter respond to ionising radiation: A steady-state radiolysis towards preparation of a 3D polymer gel dosimeter. Radiat. Phys. Chem..

[5] Kouvati K., Jaszczak M., Papagiannis P., Kadlubowski S., Wach R., Maras P., Dudek M., Kozicki M. (2019). Leuco crystal violet-Pluronic F-127 3D radiochromic gel dosimeter. Phys. Med. Biol..

[6] Muzzarelli R.A.A. (2009). Genipin-crosslinked chitosan hydrogels as biomedical and pharmaceutical aids. Carbohydr. Polym..

[7] Billiet T., Vandenhaute M., Schelfhout J., Van Vlierberghe S., Dubruel P. (2012). A review of trends and limitations in hydrogel-rapid prototyping for tissue engineering. Biomaterials.

[8] Verhulsel M., Vignes M., Descroix S., Malaquin L., Vignjevic D.M., Viovy J.-L. (2014). A review of microfabrication and hydrogel engineering for micro-organs on chips. Biomaterials.

[9] De Deene Y., Vergote K., Claeys C., De Wagter C. (2006). The fundamental radiation properties of normoxic polymer gel dosimeters: A comparison between a methacrylic acid based gel and acrylamide based gels. Phys. Med. Biol..

[10] Kozicki M., Jaszczak M., Maras P., Dudek M., Cłapa M. (2017). On the development of a VIPARnd radiotherapy 3D polymer gel dosimeter. Phys. Med. Biol..

[11] Joshi C., Schreiner L.J. (2019). High dose rate brachytherapy three-dimensional gel dosimetry using optical computed tomography readout. J. Phys. Conf. Ser..

[12] Kozicki M., Kwiatos K., Dudek M., Stempien Z. (2018). Radiochromic gels for UV radiation measurements in 3D. J. Photochem. Photobiol. A.

[13] Kozicki M., Kołodziejczyk M., Szynkowska M., Pawlaczyk A., Lesniewska E., Matusiak A., Adamus A., Karolczak A. (2016). Hydrogels made from chitosan and silver nitrate. Carbohydr. Polym..

[14] Muzzarelli R.A.A., Boudrant J., Meyer D., Manno N., DeMarchis M., Paoletti M.G. (2012). Current views on fungal chitin/chitosan, human chitinases, food preservation, glucans, pectins and inulin: A tribute to Henri Braconnot, precursor of the carbohydrate polymers science, on the chitin bicentennial. Carbohydr. Polym..

[15] Modrzejewska Z., Skwarczyńska A., Douglas T.E.L., Biniaś D., Maniukiewicz W., Sielski J. (2015). Structure of chitosan gels mineralized by sorption. J. Mol. Struct..

[16] Toppazzini M., Coslovi A., Boschelle M., Marsich E., Benincasa M., Gennaro R., Paoletti S. (2011). Can the interaction between the antimicrobial peptide LL-37 and alginate be exploited for the formulation of new biomaterials with antimicrobial properties?. Carbohydr. Polym..

[17] Paulino A.T., Guilherme M.R., de Almeida E.A.S., Pereira A.G.B., Muniz E.C., Tambourgi E.B. (2009). One-pot synthesis of a chitosan-based hydrogel as a potential device for magnetic biomaterial. J. Magn. Magn. Mater..

[18] Yan H., Dai J., Yang Z., Yang H., Cheng R. (2011). Enhanced and selective adsorption of copper(II) ions on surface carboxymethylated chitosan hydrogel beads. Chem. Eng. J..

[19] Gao J., Liu R., Wu J., Liu Z., Li J., Zhou J., Hao T., Wang Y., Du Z., Duan C. (2012). The use of chitosan based hydrogel for enhancing the therapeutic benefits of adipose-derived MSCs for acute kidney injury. Biomaterials.

[20] Yadollahi M., Farhoudian S., Namazi H. (2015). One-pot synthesis of antibacterial chitosan/silver bio-nanocomposite hydrogel beads as drug delivery systems. Int. J. Biol. Macromol..

[21] Caswell K.K., Bender C.M., Murphy C.J. (2003). Seedless, surfactantless wet chemical synthesis of silver nanowires. Nano Lett..

[22] Choi S.-H., Lee S.-H., Hwang Y.-M., Lee K.-P., Kang H.-D. (2003). Interaction between the surface of the silver nanoparticles prepared by γ-irradiation and organic molecules containing thiol group. Radiat. Phys. Chem..

[23] Hussain I., Brust M., Papworth A.J., Cooper A.I. (2003). Preparation of acrylate-stabilized gold and silver hydrosols and gold–polymer composite films. Langmuir.

[24] Nersisyan H.H., Lee J.H., Son H.T., Won C.W., Maeng D.Y. (2003). A new and effective chemical reduction method for preparation of nanosized silver powder and colloid dispersion. Mater. Res. Bull..

[25] Pan A.L., Zheng H.G., Yang Z.P., Liu F.X., Ding Z.J., Qian Y.T. (2003). Gamma-irradiation-induced Ag/SiO2composite films and their optical absorption properties. Mater. Res. Bull..

[26] Suber L., Sondi I., Matijevic E., Goia D.V. (2005). Preparation and the mechanisms of formation of silver particles of different morphologies inhomogeneous solutions. J. Coll. Interface Sci..

[27] Trapalis C.C., Vaimakis T., Kharlamov A., Kokkoris M., Kordas G., Gogotsi Y.G., Uvarova I.V. (2003). Nanostructured MeSiO_2_ (Me = Ag, Cu) coatings with antibacterial activity. Nanostructured Materials and Coatings for Biomedical and Sensor Applications.

[28] Wei D., Sun W., Qian W., Ye Y., Ma X. (2009). The synthesis of chitosan-based silver nanoparticles and their antibacterial activity. Carbohydr. Res..

[29] Atiyeh B.S., Costagliola M., Hayek S.N., Dibo S.A. (2007). Effect of silver onburn wound infection control and healing: Review of the literature. Burns.

[30] Choi J.-H., Lee S.-W., Jeong J.-H., Choi D.-G., Lee E.-S. (2009). Direct imprint of conductive silver patterns using nanosilver particles and UV curable resin. Microelectron. Eng..

[31] Klasen H.J. (2000). A historical review of the use of silver in the treatment of burns. II. Renewed interest for silver. Burns.

[32] Wei D., Qian W. (2008). Facile synthesis of Ag and Au nanoparticles utilizing chitosan as a mediator agent. Colloids Surf. B.

[33] Yang X., Wang L. (2007). Silver nanocrystals modified microstructured polymer optical fibres for chemical and optical sensing. Opt. Commun..

[34] Chen R., Chen Q., Huo D., Ding Y., Hu Y., Jiang X. (2012). In situ formation of chitosan–gold hybrid hydrogel and its application for drug delivery. Colloids Surf. B.

[35] Marsich E., Travan A., Donati I., Di Luca A., Benincasa M., Crosera M., Paoletti S. (2011). Biological response of hydrogels embedding gold nanoparticles. Colloids Surf. B.

[36] Sacco P., Travan A., Borgogna M., Paoletti S., Marsich E. (2015). Silver-containing antimicrobial membrane based on chitosan–TPP hydrogel for the treatment of wounds. J. Mater. Sci.—Mater. Electron..

[37] Tyliszczak B., Drabczyk A., Kudłacik-Kramarczyk S., Bialak-Wąs K., Sobczak-Kupiec A. (2017). In vitro cytotoxicity of hydrogels based on chitosan and modified with gold nanoparticles. J. Polym. Res..

[38] Giri T.K., Thakur A., Alexander A., Badwaik A.H., Tripathi D.K. (2012). Modified chitosan hydrogels as drug delivery and tissue engineering systems: Present status and applications. Acta Pharm. Sinic. B.

[39] Futyra A.R., Liskiewicz M.K., Sebastian V., Irusta S., Arruebo M., Stochel G., Kyziol A. (2015). Development of non cytotoxic chitosan-gold nanocomposites as efficient antibacterial materials. ACS Appl. Mater. Interfaces.

[40] Mohandas A., Deepthi S., Biswas R., Jayakumar R. (2018). Chitosan based metallic nanocomposite scaffolds as antimicrobial wound dressings. Bioact. Mater..

[41] Kozicki M., Sąsiadek E., Kadlubowski S., Dudek M., Karbownik I. (2020). Radiation sensitive polyacrylonitrile microfibres doped with PDA nanoparticles. Radiat. Phys. Chem..

[42] Kozicki M., Sasiadek E. (2011). Textile UV detector with 2,3,5-triphenyltetrazolium chloride as an active compound. Radiat. Meas..

[43] Sąsiadek E., Jaszczak M., Skwarek J., Kozicki M. (2021). NBT-Pluronic F-127 hydrogels printed on flat textiles as UV radiation sensors. Materials.

[44] Stempien Z., Khalid M., Kozicki M., Kozanecki M., Varela H., Filipczak P., Pawlak R., Korzeniewska E., Sąsiadek E. (2019). In-situ deposition of reduced graphene oxide layers on textile surfaces by the reactive inkjet printing technique and their use in supercapacitor applications. Synth. Met..

[45] Haiss W., Thanh N.T.K., Aveyard J., Fernig D.G. (2007). Determination of size and concentration of gold nanoparticles from UV-Vis spectra. Anal. Chem..

[46] Grancaric A.M., Tarbuk A., Pusic T. (2006). Electrokinetic properties of textile fabrics. Color. Technol..

[47] Luxbacher T., Pušić T., Bukšek H., Petrinić I. (2016). The zeta potential of textile fabrics: A review. Tekstil.

