# Supplementary material for: Golden and Silver–Golden Chitosan Hydrogels and Fabrics Modified with Golden Chitosan Hydrogels
Source: Int J Mol Sci. 2022 May 12;23(10):5406. doi: 10.3390/ijms23105406 (PMC9141307; doi:10.3390/ijms23105406)
Supplement: Supplementary file 1 [file ijms-23-05406-s001.zip › ijms-1702317-supplementary.pdf]

SUPPORTING INFORMATION

Golden and Silver–Golden Chitosan Hydrogels and Fabrics Modified with Golden Chitosan Hydrogels

Marek Kozicki[1,*], Aleksandra Pawlaczyk[2], Aleksandra Adamska[2], Małgorzata Iwona Szynkowska-Jóźwik[2], Elżbieta Sąsiadek-Andrzejczak[1]

[1] Department of Mechanical Engineering, Informatics and Chemistry of Polymer Materials, Faculty of Material Technologies and Textile Design, Lodz University of Technology, Zeromskiego 116, 90-543 Lodz, Poland; elzbieta.sasiadek@p.lodz.pl

[2] Institute of General and Ecological Chemistry, Faculty of Chemistry, Lodz University of Technology, Zeromskiego 114, 90-543 Lodz, Poland; aleksandra.pawlaczyk@p.lodz.pl (A.P.); adamska.grafik@gmail.com (A.A.); malgorzata.szynkowska@p.lodz.pl (M.I.S.-J.)

* Correspondence: marek.kozicki@p.lodz.pl

Abstract: Golden and silver–golden chitosan hydrogels and hydrogel-modified textiles of potential biomedical applications are investigated in this work. The hydrogels are formed by reactions of chitosan with $HAuCl_4 \cdot xH_2O$. For above the critical concentration of chitosan (c*), chitosan–Au hydrogels were prepared. For chitosan concentrations lower than c*, chitosan–Au nano- and microgels were formed. To characterise chitosan–Au structures, sol–gel analysis, UV–Vis spectrophotometry and dynamic light scattering were performed. Au concentration in the hydrogels was determined by the flame atomic absorption spectrophotometry. Colloidal chitosan–Au solutions were used for the modification of fabrics. The Au content in the modified fabrics was quantified by inductively coupled plasma mass spectrometry technique. Scanning electron microscopy with energy dispersion X-ray spectrometer was used to analyse the samples. Reflectance spectrophotometry was applied to examine the colour of the fabrics. The formation of chitosan–Au–Ag hydrogels by the competitive reaction of Au and Ag ions with the chitosan macromolecules is reported.

Keywords: chitosan-Au hydrogel; chitosan-Au microgel; chitosan-Au-Ag hydrogels; chitosan-Au modified fabrics; ICP-QMS; FAAS

1. Introduction

See the main article.

2. Materials and methods

See the main article.

3. Results and discussion

3.1. Vis spectrophotometry and DLS measurements. Micro- and nano-gels.

The solutions of chitosan reaction products with $HAuCl_4$ remaining in the wells of the 24-well cell culture template (after removing chitosan-Au

hydrogels) were measured with Vis spectrophotometry. The results obtained are presented in Supporting Figure S1.

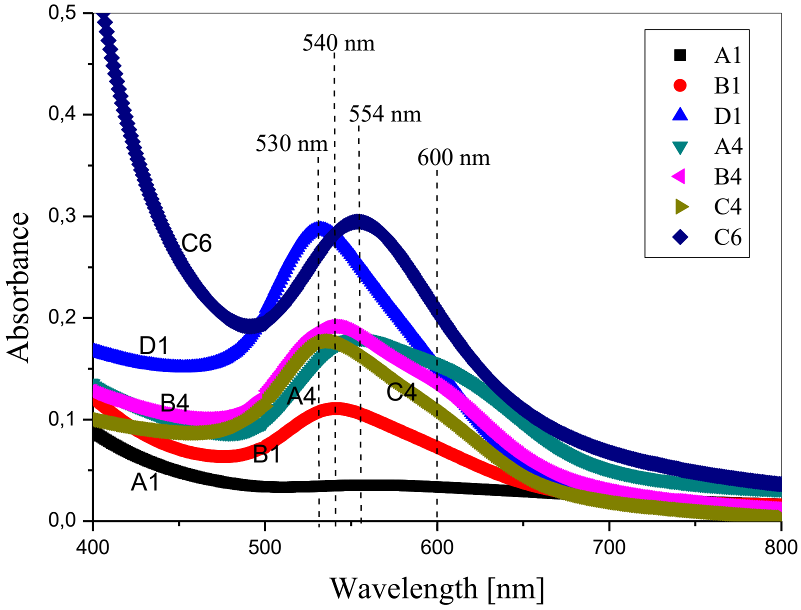

Supporting Figure S1. Absorbance spectra of Vis spectrophotometry for chitosan-Au microgel solutions. The spectra are shown for representative samples of the 2 % chitosan reaction with $HAuCl_4$ at 23°C, as follows: A1, B1 and D1 is for a 1 hour reaction with 1, 0.5 and 0.05 % $HAuCl_4$, respectively; whereas, A4, B4 and C4 is for a 22 hour reaction with 1, 0.5 and 0.1 % $HAuCl_4$, respectively. Sample C6 corresponds to a 0.5% chitosan 3-hour reaction with 1% $HAuCl_4$ at 23°C.

The solutions corresponding to the samples measured by Vis spectrophotometry were also analysed with a dynamic light scattering instrument (DLS). The results are summarised in Supporting Table S1. In all tested samples except C6, DLS measurements showed the formation of monofraction particles with a number-weighted mean hydrodynamic diameter in the range >0.8<1.0 μm. The volume-weighted results for A4 indicated a particle fraction of around 36 nm. All these observed particles are believed to be chitosan-Au microgels with probably Au particle clusters embedded in their structure. For C6, DLS number-, volume- and intensity-weighted measurements showed the formation of several particle fractions. The number weighted results showed the presence of nanoparticles with an average hydrodynamic diameter of ~1 nm. These are believed to be Au nanoparticles. However, the volume- and intensity-weighted results also showed the formation of particles with a mean hydrodynamic diameter of about 20 nm, and the intensity-weighted measurements showed the formation of particles with a diameter of about 0.1 μm. These are believed to be both Au particle clusters and chitosan-Au nanogels. Note that sample C6 was made with the lowest concentration of chitosan (0.5%) and low concentration of $HAuCl_4$ (0.1%). In this case, a wall-to-wall hydrogel was not formed; mixed ingredients remained as solution. Increasing the concentration

of chitosan to 1% resulted only in the formation of a solution, however, with an increased viscosity observed with the naked eye (main text, Table 3). A further increase in chitosan concentration to 1.5% resulted in the formation of a firm chitosan-Au hydrogel (main text, Table 3). Thus, following the observations reported in Table 3 (main text) for A6, B6 and C6 and obtained from DLS measurements for C6, it is clear that increasing the chitosan concentration promotes intermolecular cross-linking towards formation of firm chitosan-Au chitosan hydrogels. On the contrary, lowering the chitosan concentration favors the formation of micro-, nano-Au and chitosan-Au structures ($HAuCl_4$ concentration in these cases was fixed at 1%). It is assumed that the formation of nano-microgels occurs by intramolecular cross-linking of single chitosan chains or both intra- and intermolecular cross-linking of several chitosan chains, which favors the formation of larger microgels. Overall, the results obtained for the chitosan reaction with Au are consistent with those published elsewhere for chitosan-Ag nano-, micro- and macro-hydrogels [1].

Supporting Table S1. DLS measurement of selected solutions remaining after chitosan-Au nano/microgels preparation (2% chitosan): A1, B1 and D1 refers to a 1-hour reaction with 1, 0.5 and 0.05 % $HAuCl_4$, respectively; whereas, A4, B4 and C4 is for a 22-hour reaction with 1, 0.5 and 0.1% $HAuCl_4$, respectively. Sample C6 corresponds to 0.5% chitosan a 3-hour reaction with 1% $HAuCl_4$ at 23°C. Each sample was measured in duplicate. *The accuracy of the DLS instrument is one decimal place.

	A1		B1		D1		A4		B4		C4		C6	
	Mean diam. [nm]	%	Mean diam. [nm]	%	Mean diam. [nm]	%	Mean diam. [nm]	%	Mean diam. [nm]	%	Mean diam. [nm]	%	Mean diam. [nm]	%
Intensity weighted	865.4	100	866.0	100	863.9	100	842.4	100	864.8	100	866.0	100	1.1; 22.7; 112.7	14.4; 3.7; 81.9
Volume weighted	879.4	100	880.0	100	878.0	100	36.2; 859.2	64.3; 35.7	878.8	100	880.0	100	1.1; 21.1	100.0; 0.0*
Number weighted	837.5	100	838.3	100	836.1	100	808.4	100	837.2	100	838.1	100	1.0	100

3.2. Flame Atomic Absorption Spectrophotometry (FAAS) of hydrogels modified with Au

The box-whisker plot for all Au concentrations (mg/L) in all variants of modified chitosan-Au hydrogels is shown in Supporting Figure S2. The corresponding data for wet chitosan-Au hydrogels at specific swelling point is also provided in Supporting Table S2.

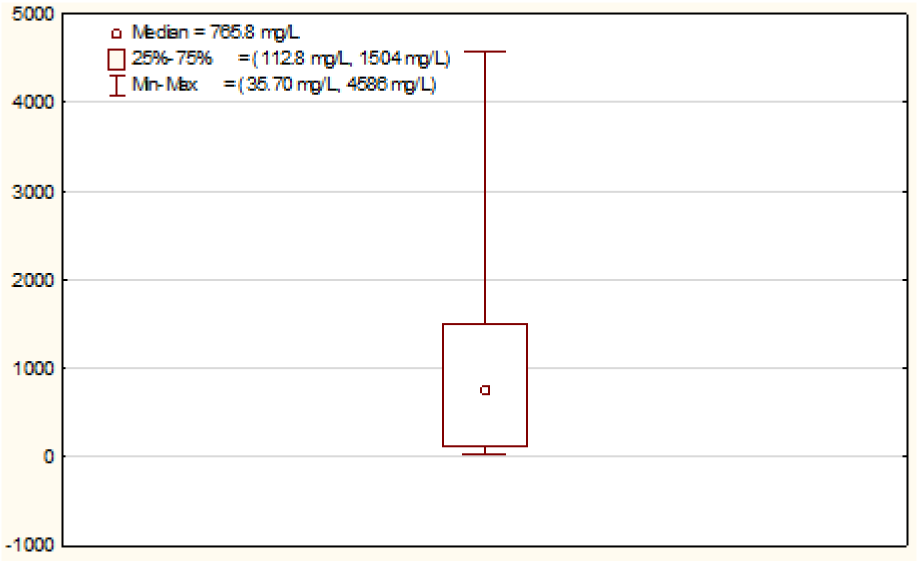

Supporting Figure S2. The box-whisker plot for all Au concentrations (mg/L) in all variants of modified chitosan-Au hydrogels.

Supporting Table S2. Descriptive statistics for Au concentration determined in wet chitosan-Au hydrogels at maximal swelling point [mg/L]

	Descriptive Statistics for Au concentration in modified hydrogels							
	Valid N	Mean	Median	Minimum	Maximum	Range	Variance	Std.Dev.
Concentration of Au [mg/L]	87	1024	765.8	35.70	4586	4550	1157990	1076

Since the null hypothesis regarding the normal distribution of Au concentration in modified hydrogels was rejected, the non-parametric Kruskal-Wallis test was employed. This test is a non-parametric alternative towards one-way ANOVA, where it can be generally assumed that the *n* independent samples are from the same population or from a population with the same median. The significance of the differences in Au concentration in the hydrogels between the formed groups was assessed in relation to four different parameters such as:

1) initial concentration of chloroauric acid, $HAuCl_4$ (4 groups were studied: I with 1.0% of $HAuCl_4$ (n=28); II with 0.5% of $HAuCl_4$ (n=20); III with 0.1% of $HAuCl_4$ (n=19) and IV with 0.05% of $HAuCl_4$ (n=20));
2) contact time (5 groups were distinguished: I with contact time 1 h (n=15); II with contact time 2 h (n=16); III with contact time between 3 and 4 h (n=24); IV with contact time 22 h (n=16) and V with contact time 46 h (n=16));
3) swelling time (5 groups were chosen for the analysis: I with swelling time between 15 and 60 min (n=34); II with swelling time in a range of 80–245 min (n=32); III with a swelling time between 250–270 min (n=4); IV with a swelling time within 275–330 min range (n=13) and V with the swelling time between 1330 and 1350 min (n=4));
4) initial concentration of chitosan (only 3 groups were considered: I with chitosan concentration of 2% (n=79, the inter- and intra-individual

variation within this particular group was latter investigated by PCA method); II with 1.5% of chitosan (n=4) and III with 1% of chitosan (n=4)).

The potential impact of all these four parameters on the Au concentration in chitosan-Au hydrogels was then individually tested. Quantitative parameters such as swelling time, contact time, $HAuCl_4$ concentration and initial chitosan concentration were coded, expressed in certain value ranges and presented as groups in the box-whisker plots in order to fully examine their distribution in the test groups.

In the case of the influence of $HAuCl_4$ initial concentration, a predictable increase in Au concentration in hydrogels was observed along with an increase in $HAuCl_4$ concentration (Supporting Figure S3A), as median Au values in hydrogels decreased towards lower $HAuCl_4$ concentrations. The greatest differentiation of the most typical observations was reported for the results with the lowest concentration of $HAuCl_4$, contrary to group IV with less significant variability of Au concentration in the whole group. At the same time, the biggest variation within the 25% of the highest values were observed for groups II and III determined by the concentration of $HAuCl_4$ of 0.1% and 0.5%, respectively. It can be additionally concluded that with the increase of $HAuCl_4$ concentration, the reproducibility of the modified hydrogel formation probably also increases and smaller deviations in the data for Au concentration are noted.

Based on multiple comparisons of the mean ranks for all the analyzed groups, statistically significant differences were confirmed only between the pairs: group I and III (1.0% and 0.1% of $HAuCl_4$, respectively); between I and IV (1.0% and 0.05% of $HAuCl_4$, respectively); between group II and III (0.5% and 0.1% of $HAuCl_4$, respectively) and finally between group II and IV (0.5% and 0.05% of $HAuCl_4$, respectively). There were no statistically significant differences between the pairs: group I and II and groups III and IV, for which the difference in $HAuCl_4$ concentration was only two-fold. For the remaining groups for which statistically significant differences in Au concentration in hydrogels were confirmed, the difference in $HAuCl_4$ concentration was at least 5 times greater. Hence, it can be concluded that a twofold change in $HAuCl_4$ concentration does not lead to a statistically significant increase in gold concentration in the hydrogel sample. Only a 5-fold (or more) increase in $HAuCl_4$ concentration can contribute to a significant increase in Au concentration in hydrogels.

In the case of the influence of contact time (Supporting Figure S3B), no statistically significant differences were found between the analysed groups. In general, it can be assumed that the level of the retained gold in hydrogels also increases as the contact time increases. The smallest variation of results for both 50% of the most typical values and 25% of the highest results was noted in group I with the shortest contact time of 1 h, while the greatest variation in the most typical observations was seen for the last group (with the longest contact time between reagents – 46 hours). Moreover, in this group the highest value of the median was determined.

An overall reverse trend was observed for the effect of the swelling time (Supporting Figure S3C). In the case of this parameter, it can be assumed that the increase in the swelling time contributed to the reduction of the residual gold concentration in hydrogels. The largest differences in the concentration

of gold for 50% of the most typical values concerned groups with the shortest swelling time (group I and II), however, no statistically significant differences were found in Au concentration in hydrogels in relation to this criterion. Within both groups (I and II), the highest concentrations of Au were found in the entire study population.

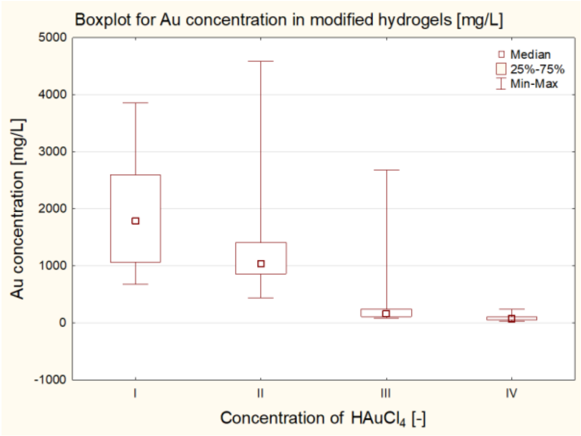

A

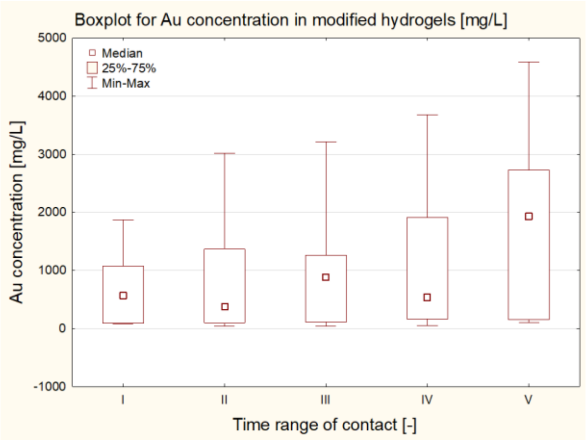

B

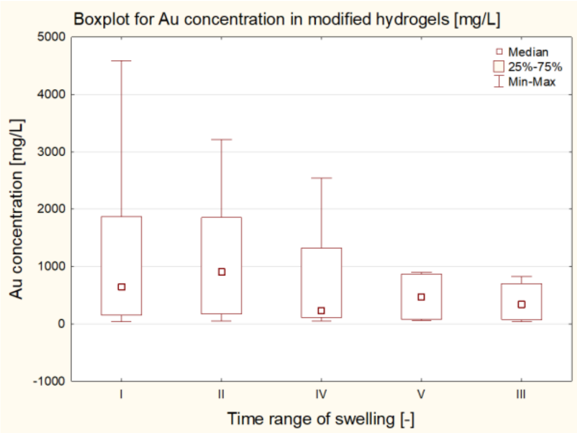

C

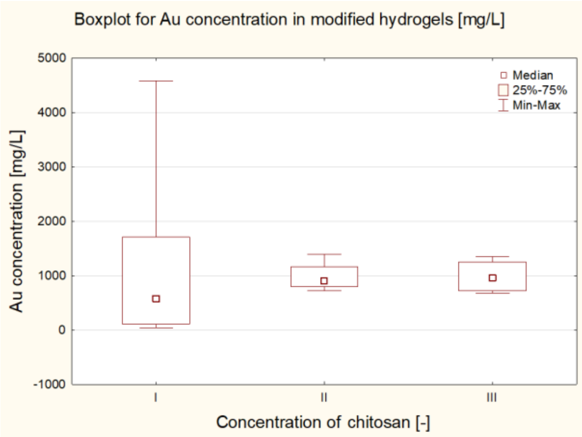

D

Supporting Figure S3. A: Box-whisker plot for all Au concentration in chitosan-Au hydrogels in relation to the initial concentration of $HAuCl_4$, where group I is 1.0% $HAuCl_4$; group II is 0.5% $HAuCl_4$, group III is 0.1% $HAuCl_4$ and group IV is 0.05% $HAuCl_4$. B: Box-whisker plot for all Au concentrations in modified hydrogels in relation to the selected contact time between the reagents (chitosan and Au), where group I is for the contact time 1 h; group II is for the contact time 2 h; group III is for the contact time between 3 and 4 h; group IV is for the contact time 22 h, and group V is for the contact time 46 h. C: Box-whisker plot for all Au concentrations in chitosan-Au hydrogels in relation to chosen swelling time ranges, where group I is for the swelling time between 15 and 60 min; group II is for the swelling time between 80 amd 245 min; group III is for the swelling time between 250 and 270 min; group IV is for the swelling time between 275 and 330 min and group V is for the the swelling time between 1330 and 1350 min. D: Box-whisker plot for all Au concentrations in chitosan-Au hydrogels in relation to the initial concentration of chitosan, where group I is for the chitosan concentration of 2%; group II is for 1.5% of chitosan and group III is for 1% of chitosan.

The influence of the initial chitosan concentration was also verified (Supporting Figure S3D) and no statistically significant differences were found in Au levels in the range of the tested chitosan concentrations. However, it should be emphasized that this conclusion may be influenced by the small number of observations for groups II and III (four each). At the same time, it is clearly visible that the greatest variability in the data (both within the typical values and within the 25% of the highest observations) was demonstrated by the data collected for the highest initial concentration of chitosan.

The PCA method was employed to analyze the inter- and intra-individual variation within the studied population of 79 objects with the same initial chitosan concentration of 2% (the variants with 1.5% and 1% chitosan were rejected due to low number of representatives; 4 for each group) (Supporting Figure S4). Four variables were finally selected: the determined Au concentration in modified hydrogels (range from 35.70 mg/L to 4586 mg/L), initial concentration of $HAuCl_4$ (only four options were considered: 0.05%, 0.1%, 0.1% and 1% of $HAuCl_4$), swelling time (the lowest point included was 15 min while the highest was 1350 min) and the contact time (1, 2, 4, 22 and 46 h). The projection of variables on the factor plane for the first two components revealed that overall percentage of the explained variability exceeds 75%. For this reason, the analysis has been reduced to only the first two main components. It was shown that only one variable was negatively correlated with the first and second components (swelling time and contact time, respectively). The Au concentration in hydrogels and $HAuCl_4$ concentration were most strongly linked with the first component, whereas the other two variables (contact and swelling time) were related to the second component. The close location of the vectors belonging to the Au concentration in hydrogel and the concentration of $HAuCl_4$ suggests that these two variables are strongly correlated with each other (Supporting Figure S4A). Moreover, there is no correlation between contact time and $HAuCl_4$ concentration. There is clearly a negative relationship between the swelling time and the contact time of the reagents, which leads to the fact that increasing the contact time reduces the swelling time.

In order to identify the samples included in each cluster, the same graph was used in further analysis after changing the scale (Supporting Figure S4B). As a result, it was possible to trace factors, which may be responsible for the grouping of the samples. Five clusters have been distinguished. Each cluster was characterized by 2–3 parameters that clearly defined belonging to each group.

The four samples that are circled in green are those with the longest swelling time (all > 1300 min) and relatively short contact time (between 1 and 2 h). We can also observe a decrease in $HAuCl_4$ concentration from the bottom to the top of the cluster. The samples belonging to one cluster marked in red have a relatively low Au level determined in hydrogels (within the range of 35 mg/L and 301 mg/L) and, at the same time, a low concentration of the gold precursor ($HAuCl_4$ concentration ranged from 0.05% to 0.1%). It can also be seen that the beginning of this cluster is formed by the samples with the shortest contact time (1 h), while the tail is formed by the samples with the longest contact time, reaching 46 h. All samples in the blue cluster can be described with the same $HAuCl_4$ concentration of 0.5% and an Au concentration range from 444 mg/L (top of this cluster) to 1242 mg/L (bottom

of this cluster). The samples in the purple cluster can be defined by the same initial concentration of the gold precursor of 0.5% $HAuCl_4$, the very long contact time (mostly 46 h) and the Au concentration in hydrogels mainly above 2 mg/L. The samples in the cluster marked in orange have an initial concentration of $HAuCl_4$ of 1% and the highest determined Au levels in the hydrogels among all groups studied ranging from 976 mg/L (top part) to 4585 mg/L (bottom part). When analyze the location of four clusters (red, blue, purple and orange), several other features can be noticed. The samples in the red cluster have the lowest levels of Au in hydrogels and the lowest concentration of $HAuCl_4$ as opposed to the samples in the orange cluster circle.

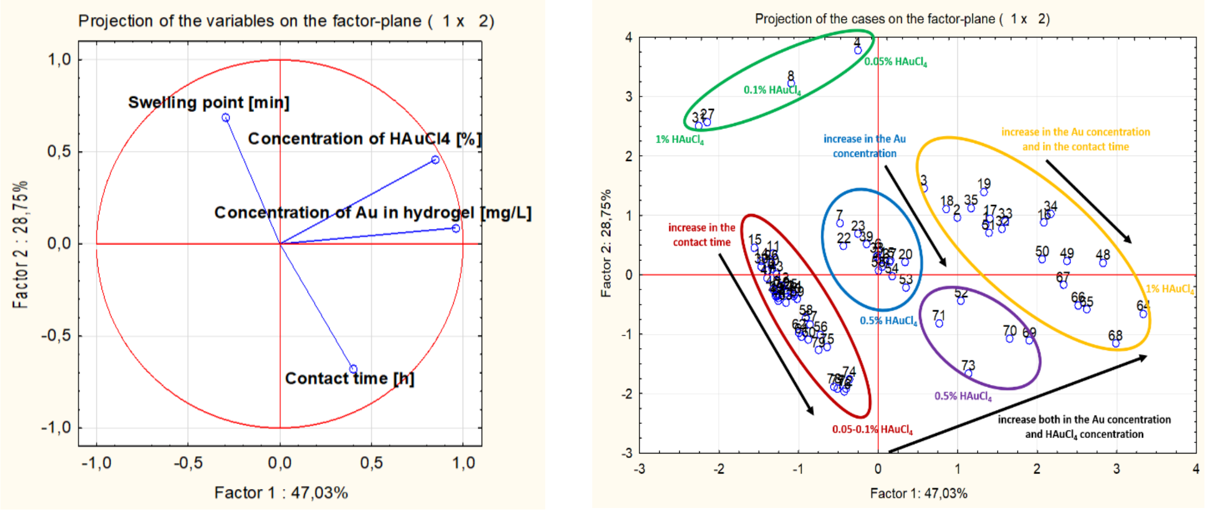

A B

Supporting Figure S4. A: The PCA projection of the variables on the first two components. B: The PCA projection of the cases on the first two components (after the scale change).

3.3. Modification of textiles – reflectance analysis.

The results of reflectance measurements for textile samples modified with colloidal solutions of chitosan-Au hydrogels are presented in Supporting Figure S5.

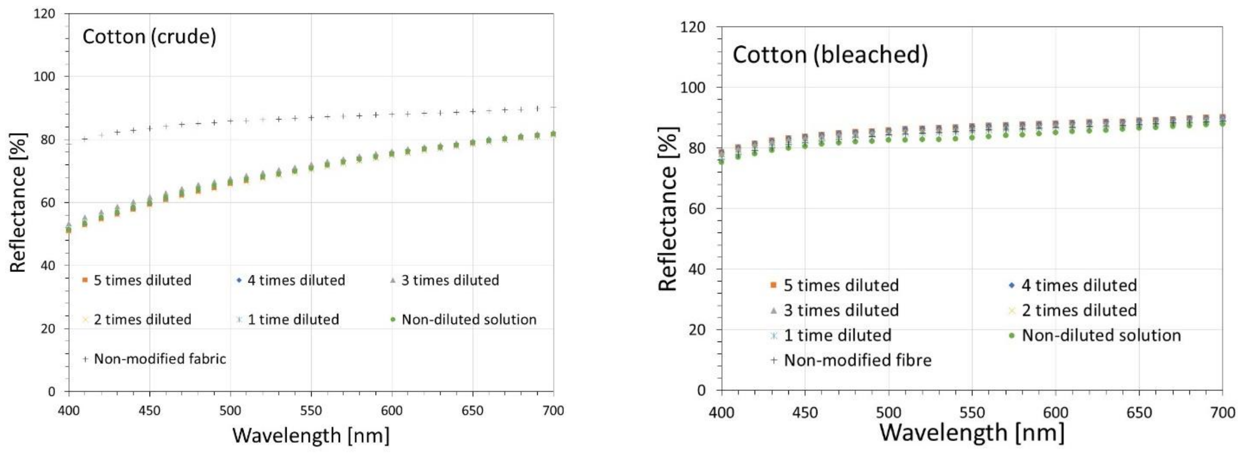

Supporting Figure 5.

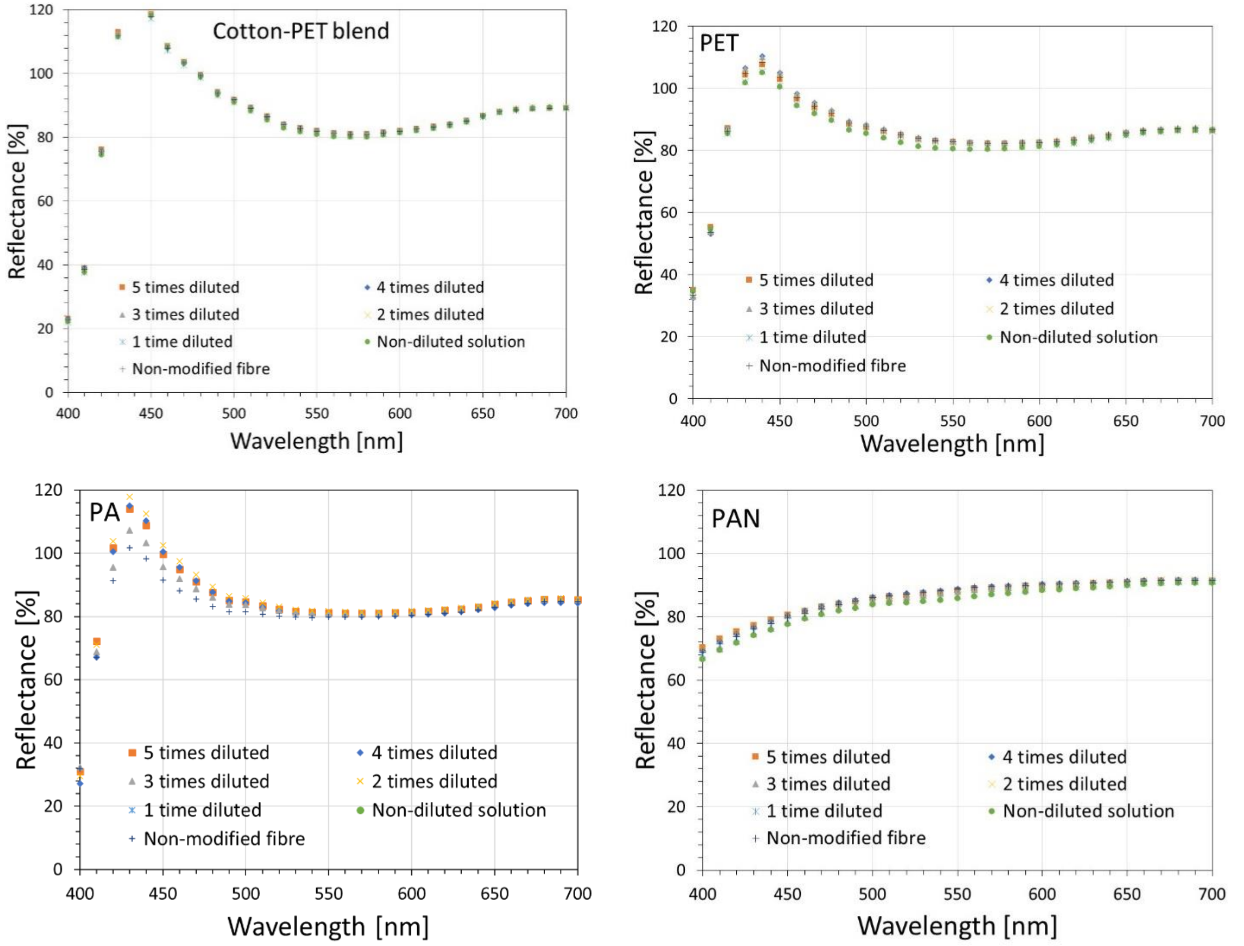

Supporting Figure S5. Reflectance spectra of cotton (crude and bleached), cotton-PET blend, PET, PA and PAN textiles modified with colloidal chitosan-Au solutions. The spectra are shown for textiles unmodified with colloidal solutions and textiles modified with solutions: undiluted and obtained after 1–5 times dilutions. Colloidal chitosan-Au solutions were prepared after a 24 h reaction time between 2% chitosan and 0.1% $HAuCl_4{\cdot}xH_2O$.

3.4. SEM analysis of the modified textiles

The morphology of textile samples modified with colloidal solutions of chitosan-Au hydrogel has been analysed with SEM technique. The results are presented in Supporting Figure S6.

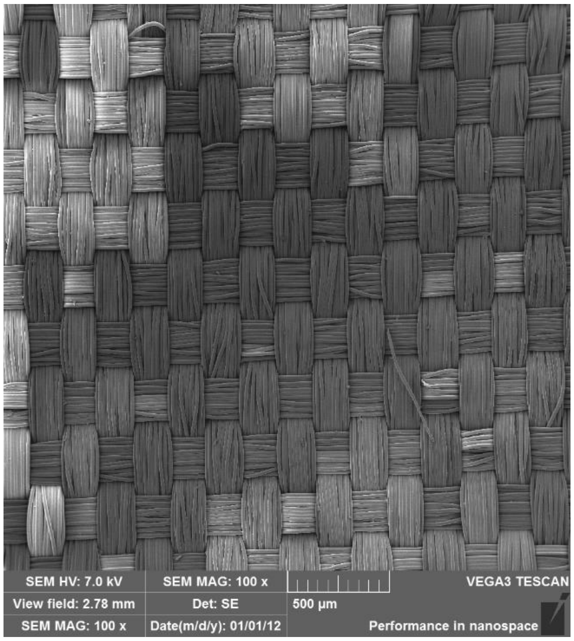

A (PET)

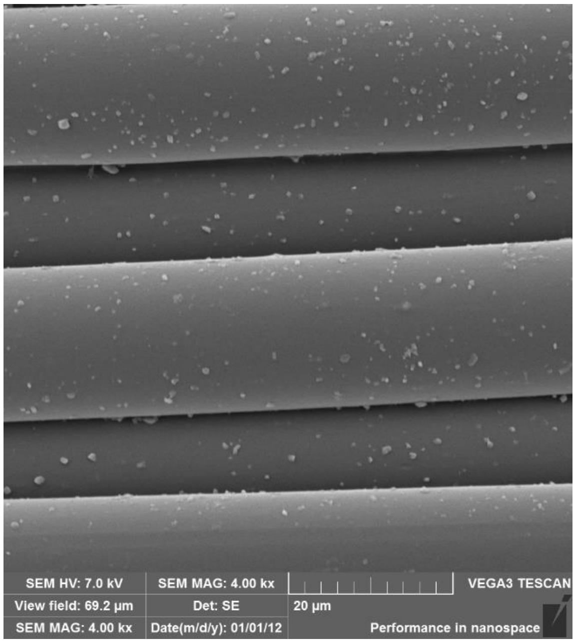

B (PET)

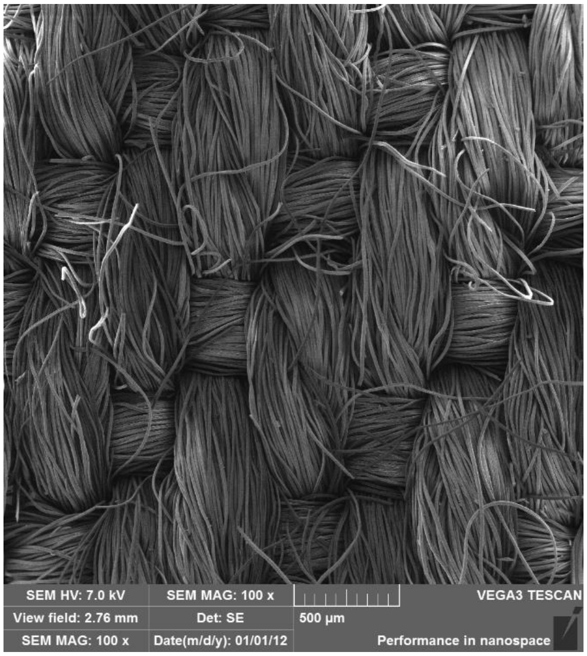

C (PAN)

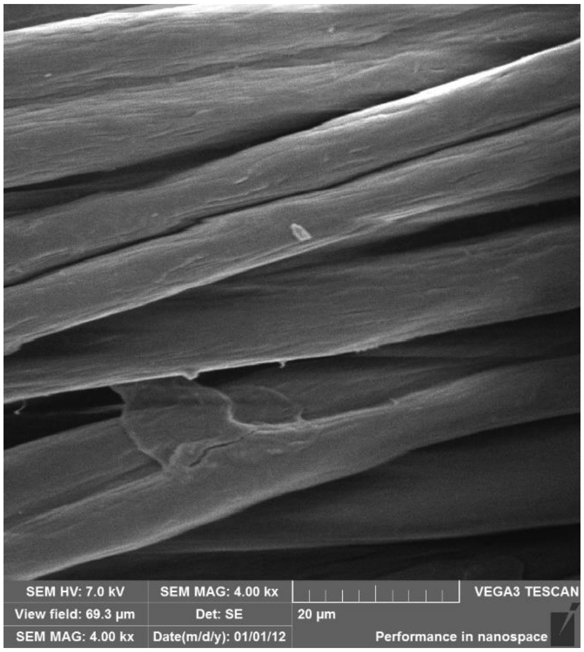

D (PAN)

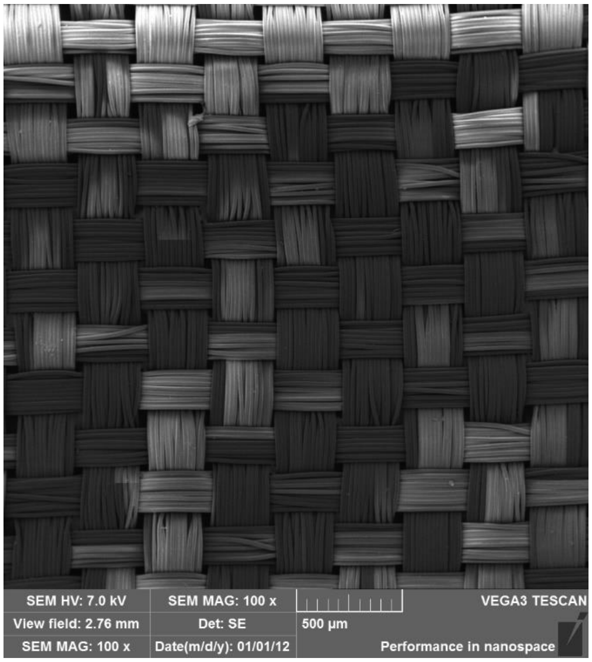

E (PA)

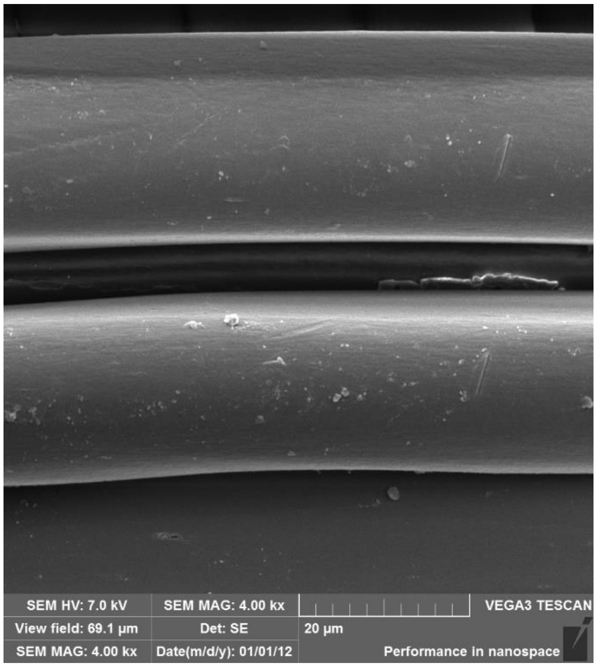

F (PA)

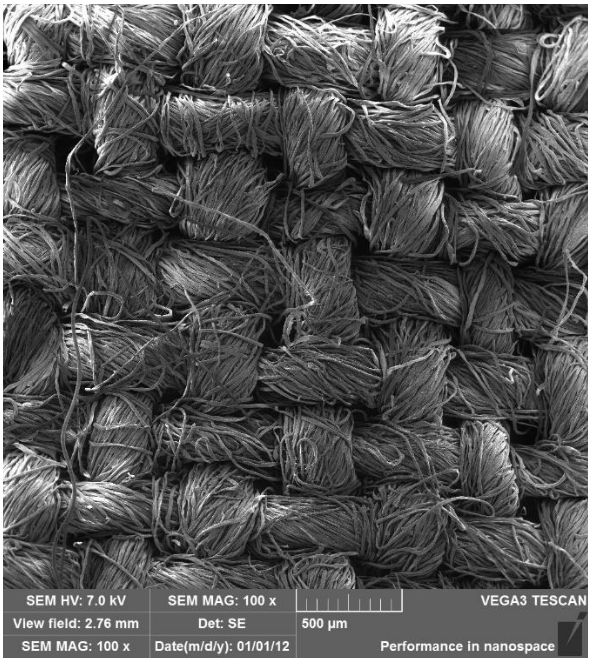

G (Cotton (bleached))

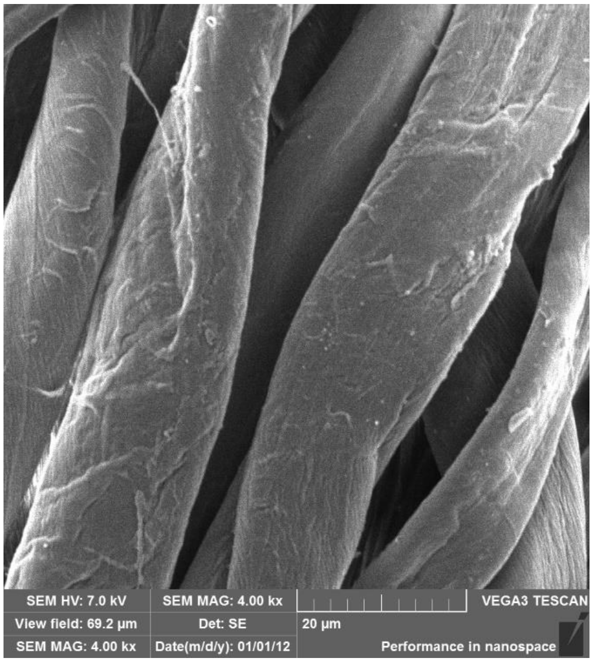

H (Cotton (bleached))

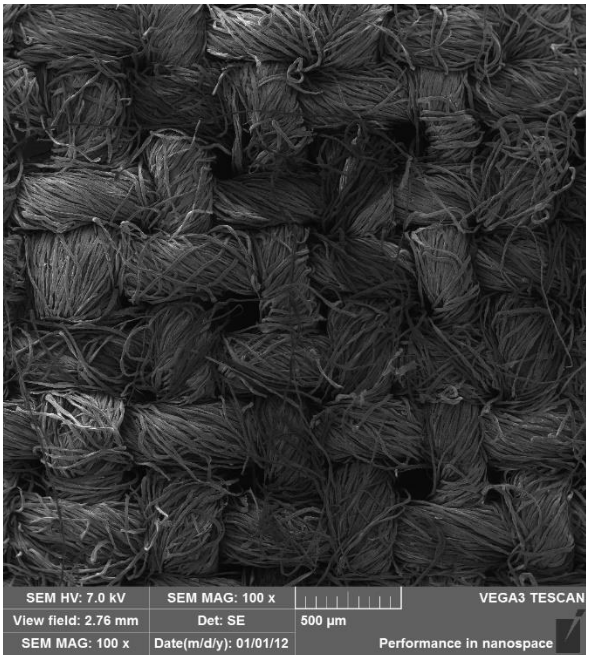

I (Cotton crude)

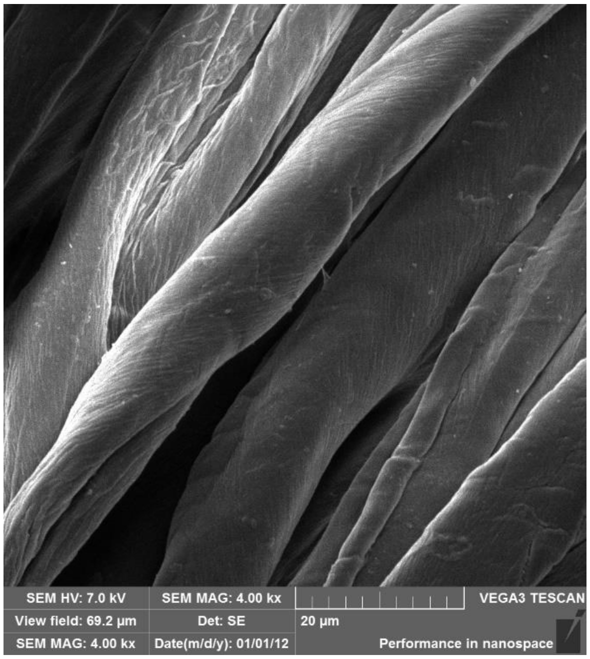

J (Cotton crude)

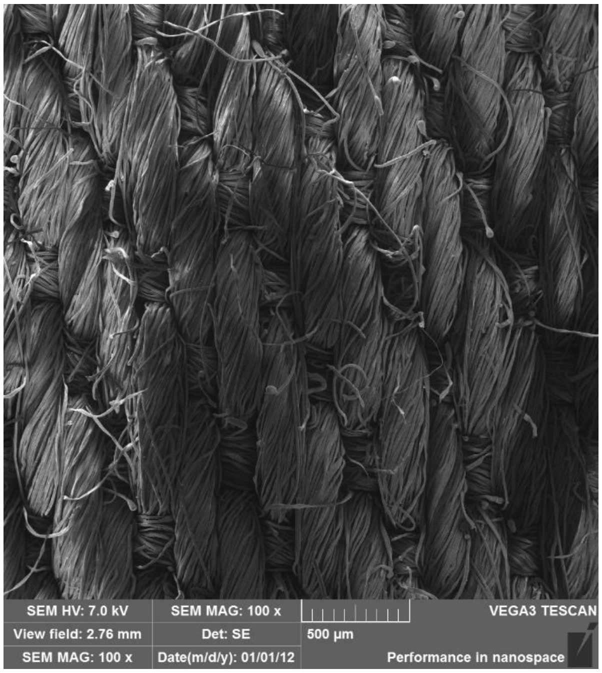

K (Cotton-PET)

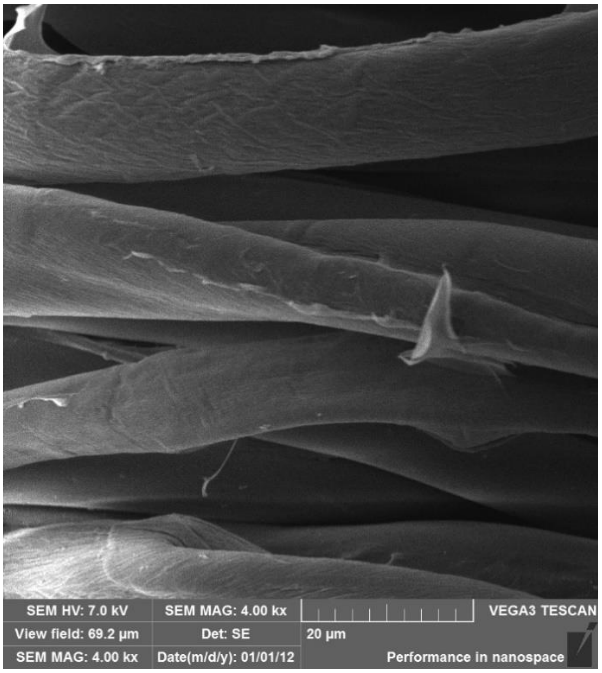

L (Cotton-PET)

Supporting Figure S6. Scanning electron microscopy analysis of textile modified with chitosan-Au colloidal solutions. Magnifications: ×100 (left column) and ×4000 (right column): PET (A–B), PAN (C–D), PA (E–F), Cotton bleached (G–H), Cotton crude (I–J), Cotton-PET (K–L). The samples were modified by padding-squeezing-drying (at 23 °C) method. The modification was performed with a colloidal solution based on a 24-h reaction between 2% chitosan and 0.1% $HAuCl_4{\cdot}xH_2O$ (undiluted solution).

3.5. ICP-MS of the modified textiles

The statistical analysis of the ICP-MS results (Kruskal-Wallis test) for Au deposited on textile samples by padding-squeezing-drying was performed as described in Experimental section (main text). The results in form of a box-whisker plot are shown in Supporting Figure S7. The box-whisker plot depicting the quantitatively measured Au concentration in different fabrics showed a large variation in the measured Au concentration levels deposited on the fabrics. The median value (10905 μg/L) was significantly different from the mean value (28628 μg/L), because the mean value was strongly affected by the highest observations obtained in this study (the maximum achieved concentration was 7 times higher than the mean value and over 18 times higher than the median value) (Supporting Table S3). It should also be emphasized that the greatest variability in the data was noticed within the 25% of the highest observations. The median (marked as a square inside the box) was visibly directed towards the lowest values (lower frame of the box) obtained in this study. Most of the concentrations recorded (75% of the data located within the 25% of lowest values and within the 50% of the most typical values) was in the range of < DL (limit of detection) up to 43305 μg/L. For only 12 samples (not treated with gold-modified hydrogels), no traces of gold were detected. Only for 5 samples the quantified Au concentration exceeded the value of 100 mg/L. In all cases, these were fabrics modified with undiluted hydrogels (after their homogenization), and most of them were cotton fabrics (bleached or crude).

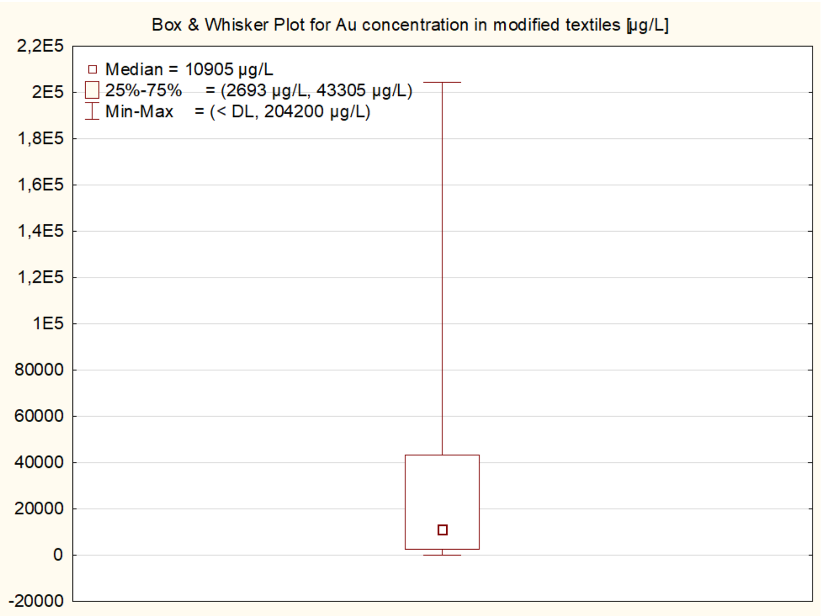

Supporting Figure S7. Box-whisker plot for all Au concentrations in all variants of modified textiles.

Supporting Table S3. Descriptive statistics for Au concentration determined in modified textiles [μg/L].

	Valid N	Mean	Median	Minimum	Maximum	Range	Variance	Std. Dev.
Concentration of Au in textiles [μg/L]	84	28628	10905	< DL	204200	204200	$1.65 \cdot 10^9$	40661

The results of measurements of gold content in samples of textiles modified with homogenized solutions of chitosan-Au hydrogels (based on the initial concentration of 0.1% $HAuCl_4$) were assessed for two variants of forming time: 24 and 48 h depending on the factors included in this study with nonparametric tests. The following were taken into account:

1) The contact time between both reagents, namely chitosan and chloroauric acid. In this case, two groups were taken into account: group I with a contact time of 24 h (n=42) and group II with 48 h contact time (n=42);
2) A specific type of fabric, which included 6 groups: PA – modified and unmodified polyamide (n=14); BC – modified and unmodified bleached cotton (n=14); RC – modified and unmodified crude (raw) cotton (n=14); C/PET modified and unmodified cotton/polyester (n=14); PAN – modified and unmodified polyacrylonitrile (n=14); PET – modified and unmodified polyester (n=14);
3) General type of textile, which included 2 groups: P – the materials without cotton or a blend of cotton with a synthetic fabric (C/PET; PA; PET; PAN; n=56) and C – cotton based materials (BB – bleached cotton and RC – crude (raw) cotton; n=28);
4) Dilution factor. A total 7 groups were distinguished, as follows: NMT – non modified textiles (n=12); T0 – textiles modified with primary homogenized hydrogels without their dilution after 24 and 48 h contact time between reagents (n=12); T2 – textiles modified with 2-fold diluted homogenized hydrogels after 24 and 48 h contact time between reagents (n=12); T4 – textiles modified with 4-fold diluted homogenized hydrogels after 24 and 48 h contact time between reagents (n=12); T8 – textiles modified with 8-fold diluted homogenized hydrogels after 24 and 48 h contact time between reagents (n=12); T16 – textiles modified with 16-fold diluted homogenized hydrogels after 24 and 48 h contact time between reagents (n=12); T32 – textiles modified with 32-fold diluted homogenized hydrogels after 24 and 48 h contact time between reagents (n=12).

The employed grouping allowed for the coding of the results and assessment of the influence of the tested factors (such as the type of fabric, dilution factor or contact time of reagents) on the total concentration of gold in modified textiles after air drying. In the case of the contact time, no statistically significant differences were found between analyzed groups (Supporting Figure S8A). Generally, it can be assumed that the contact time had no effect on the amount of Au contained in the modified textiles. In the box-whisker plot, the medians of both variants and the overall variance within the studied populations were comparable. These results are consistent with the data obtained for quantitative measurements of Au concentrations in hydrogel samples used for textile modification. As the same types of textiles were analyzed in both groups and the results within the groups

differed significantly, it can also be concluded that the type of fabric (not the contact time) may play an important role in this respect.

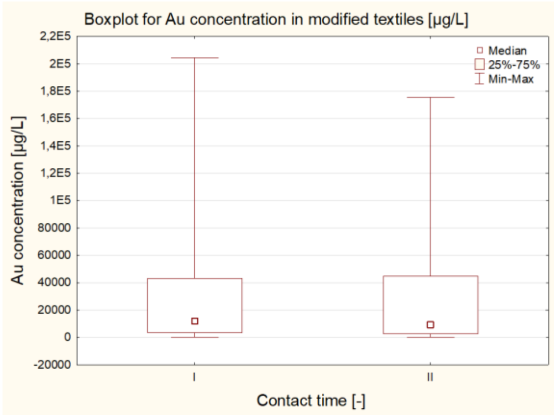

A

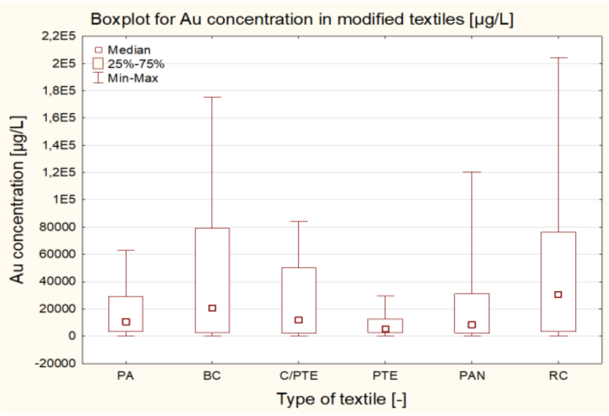

B

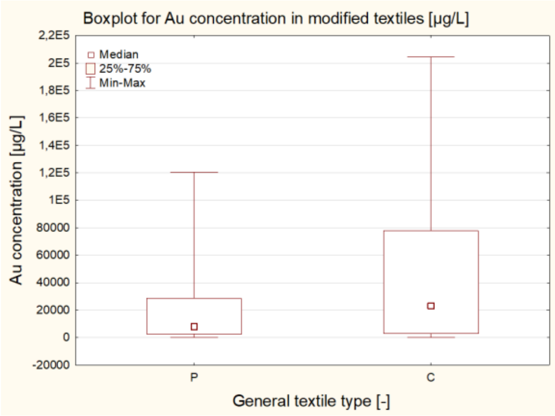

C

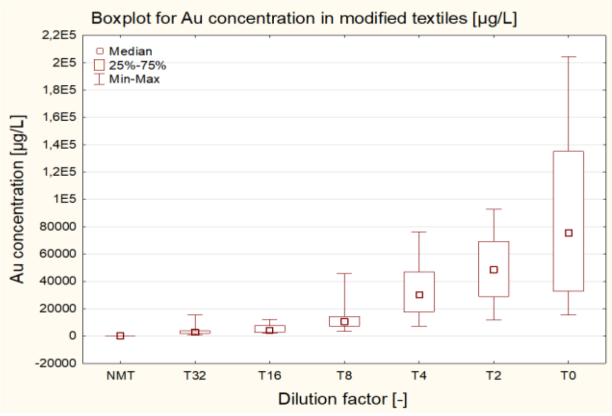

D

Supporting Figure S8. A: Box-whisker plot for all Au concentrations in the chitosan-Au colloid modified textiles in relation to the contact time between 2% chitosan and 0.1% $HAuCl_4{\cdot}xH_2O$ during the formation of chitosan-Au hydrogels from which the colloid was made, where group I refers to 24 h and group II to 48 h. B: Box-whisker plot for all Au concentrations in the modified textiles in relation to the specific type of the modified textile, where PA – modified and unmodified polyamide; BC – modified and unmodified bleached cotton; RC – modified and unmodified crude (raw) cotton; C/PET – modified and unmodified cotton/polyester; PAN – modified and unmodified polyacrylonitrile; PET – modified and unmodified polyester. C: Box-whisker plot for all Au concentrations in the modified textiles in relation to the general type of the modified textile, where P – synthetic polymer based textiles (C/PET; PA; PET; PAN) and C – cotton based materials (BB – bleached cotton and RC – crude (raw) cotton). D: Box-whisker plot for all Au concentrations in the modified textiles versus the dilution factor related to the dilutions of the initial chitosan-Au colloidal solution, which was used for textiles modification, where NMT – non modified textiles; T0, T2, T4, T8, T16, T32 – textiles modified with initial chitosan-Au colloid without dilution, 2-fold, 4-fold, 8-fold, 16-fold and 32-fold dilution of the colloid that was made from chitosan-Au hydrogel after 24 and 48 h contact time between chitosan and Au reagents.

The type of fabric was examined as a second factor. It was shown that the lowest differences in the results within the entire population were found for PET textiles, while the highest for cotton textiles (the group of crude RC cotton and bleached BC cotton), for which the highest median values were also recorded. Natural fibres contained the highest concentration of Au after deposition of the chitosan-Au colloid by padding-squeezing method among all analyzed groups with regard to the median value, mean value and individual results. The relatively high variability of data for the majority of the studied groups is related to the fact that the textiles were treated with hydrogels of different dilution factors.

Based on the median Au concentration in each of the studied textile groups (Supporting Table S4), the following sequence can be formulated: PET > PAN > PA ≈ C/PET > BC > RC. This clearly shows that cotton/PET (C/PET) group can also have a relatively high Au content, apparently due to the presence of cotton in this blend. There were no statistically significant differences in the gold concentrations in the textiles depending on the type of textile under consideration. However, due the smallest scatter of results in the PET group, it can be assumed that this textile is quite resistant to hydrogel modifications. The greatest potential for hydrogel modification was observed in the case of the cotton textiles (bleached and crude), therefore the next grouping was performed after dividing the data between two groups: cotton based and others.

The results of the nonparametric test also revealed the existence of statistically significant differences between the concentration of gold in the textiles and the general type of textile used (Supporting Figure S8C). Although twice as many observations were included in the group of polyester-based materials, the variation of both 50% of the most typical values, the median values, and the variation of the 25% of the highest scores were significantly smaller compared to the cotton-based group (Supporting Table S5). The median value for the group consisting of natural textiles (group "C") was about 3 times higher than for the synthetic textiles. The obtained data suggest that the group of synthetic textiles (PA, PET, C/PET, PAN) has a much lower ability to absorb fluids/modified hydrogels with gold than natural materials i.e. crude or bleached cotton. Cotton products possesses very good hygroscopicity, i.e. the ability to bind water, and therefore they are characterized by better susceptibility to modification with chitosan hydrogels. In general, it can be concluded that natural fibres are more prone to Au accumulation from chitosan-Au colloidal solution than synthetic fibres. The results also support the conclusion that the high dispersion of particles on the surface of polyester fibers is due to their compact structure and low surface wettability. As a result, Au particles do not penetrate the entire volume of the material, but rather remain on the surface, which can be observed during SEM measurements in the case of PET fabric.

The last parameter tested was the dilution factor. Not surprisingly, there was a strong correlation between the Au concentration in the textiles and the degree of dilution of the initial chitosan-Au colloid. As expected, the Au content decreases with increasing number of dilutions (Supporting Figure S8D). The highest Au concentrations in textiles were recorded for the materials modified with the original colloidal solution of the hydrogels (for both 24 and 48 h of contact time between the chitosan and Au reagents). No

traces of gold were detected in all textiles not treated with the colloidal hydrogel solutions. Statistically significant differences were observed between some specific groups of fabrics modified with different dilutions of homogenized hydrogel solutions. Based on the analysis of the multiple comparisons of mean ranks for all groups, it was shown that most statistically significant differences occurred between the following pairs: T0 and T16; T0 and T32; T0 and NMT; T2 and T16; T2 and T32; T2 and NMT; T4 and T32; T4 and NMT; T8 and NMT (Supporting Figure S8). These groups were characterized by at least 8 times greater dilution of the base colloidal hydrogel solutions. There was no statistically significant difference in Au concentration in the textiles between the following pairs of groups: T0 and T2; T0 and T4; T0 and T8; T2 and T4; T2 and T8; T4 and T8; T4 and T16; T8 and T16; T8 and T32; T16 and T32; and T32 and NMT. Summing up, it can be concluded that 4-fold (and smaller) dilution of chitosan-Au colloids did not cause a statistically significant decrease in the concentration of gold in the modified fabrics. On the contrary, a dilution of at least 8-fold contributed to a significant reduction in the gold concentration. The obtained results also confirmed the expected tendency - along with the increase in the number of dilutions of the initial colloidal chitosan-Au solution, the amount of retained gold decreased in the fibers of the studied textiles. Moreover, it can be noticed that among all the studied groups, for the considered parameter (dilution factor), the largest scatter of the results in the range of typical values and the highest individual results for Au concentrations were noted for the textiles treated with the initial colloidal solution of chitosan-Au (group T0). As this group consists of fabrics with different ability to absorb and retain golden hydrogel colloids, such high variability is a clear reflection of their different properties. Further to this, PCA analysis was performed (Supporting Figure S9).

Supporting Table S4. Descriptive statistics for Au concentration determined in the chitosan-Au modified textiles [μg/L] in reference to the type of the modified textile.

Textile type	Valid N	Mean	Median	Minimum	Maximum	Range	Variance	Std. Dev.
PA	14	17050	10601	< DL	62850	62849	325043654	18029
BC	14	45198	20350	< DL	175285	175284	$3.06 \cdot 10^9$	55287
C/PET	14	26520	11650	< DL	83856	83855	830681452	28821
PET	14	8548	5245	< DL	29615	29615	73842485	8593
PAN	14	22623	8197	< DL	120459	120458	$1.06 \cdot 10^9$	32519
RC	14	51831	30607	< DL	204200	204199	$3.71 \cdot 10^9$	60944

Supporting Table S5. Descriptive statistics for Au concentration determined in the chitosan-Au modified textiles [μg/L] in reference to the main type of the modified textile. P denotes the fabrics made of synthetic polymer and a cotton-polyester blend; C denotes cotton based textiles.

Textile main type	Valid N	Mean	Median	Minimum	Maximum	Range	Variance	Std. Dev.
P	56	18685	7909	< DL	120459	120458	586989229	24228
C	28	48515	23188	< DL	204200	204199	$3.27 \cdot 10^9$	57197

All information collected for the modified and unmodified textiles was further used to perform the PCA analysis. A limited number of variables were used in this analysis, because the studied textiles were defined only by the Au concentration, type of fabric, contact time of reagents during the chitosan-Au hydrogel formation or the dilution factor of homogenized chitosan-Au initial colloidal solution. In this case the total explained variability for the first two component reached 66.2% (Supporting Figure S9). The analysis revealed that the samples were clearly separated into two clusters, the main criterion of which was the general type of fabric (natural vs. synthetic). Within each cluster, the samples were sorted by the same number of dilutions of the initial colloidal solution. In addition, it can be noticed that the samples were grouped into rows according to the central value (mean/median) related with the gold level in individual groups of fabrics, so that when moving from left to right PCA graph, the samples belonging to the following groups can be distinguished: RC, BC, C/PET, PA, PAN and PET. However, again, the contact time of the reagents used for preparation of chitosan-Au hydrogel following by formation an initial colloidal chitosan-Au solution had no effect on the projection of cases onto the factor plane, as were the conclusions drawn from the non-parametric test results.

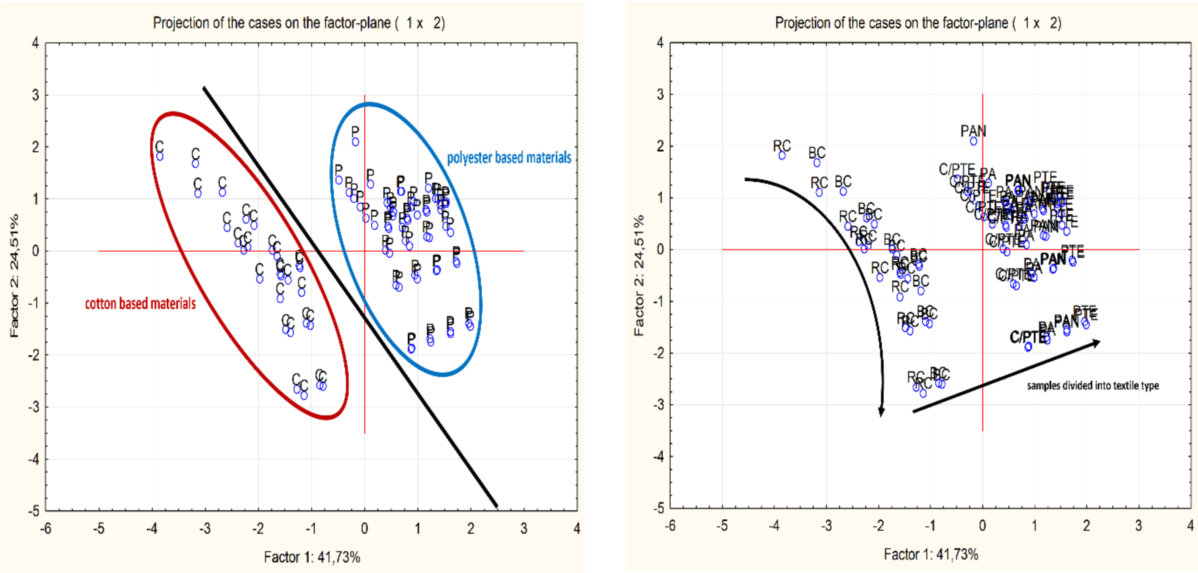

A B

Supporting Figure S9.

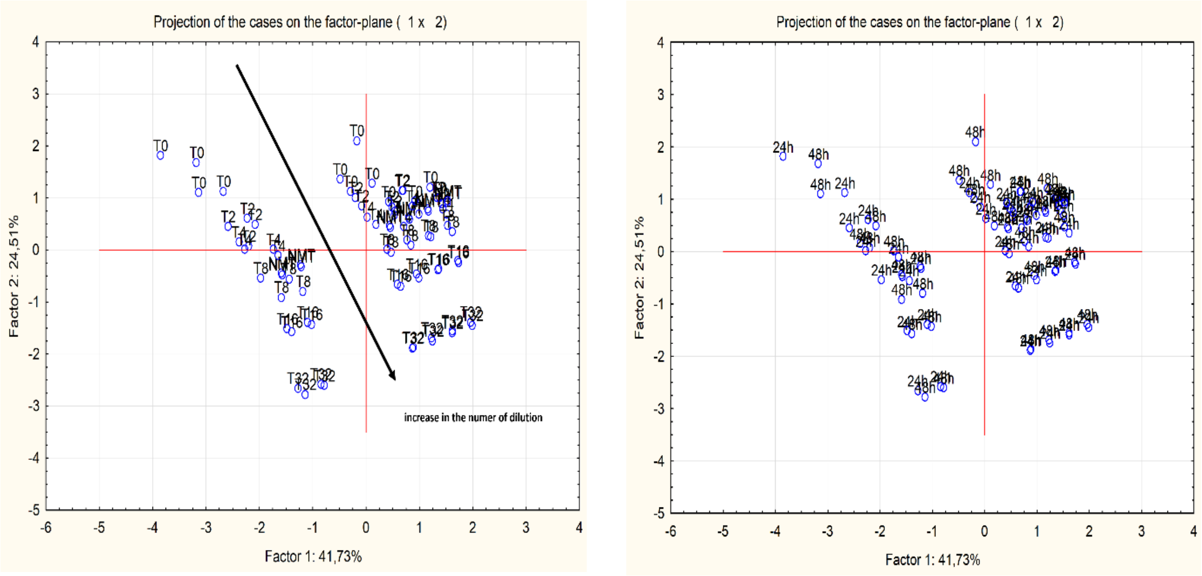

C D

Supporting Figure S9. The PCA projection of the cases on the first two components in relation to studied parameters: general textile type (A), specific textile type (B), dilution factor (C), and contact time (D).

3.6. Chitosan-Au-Ag hydrogels

The two instances of chitosan-Au-Ag formation were investigated by reacting the chitosan solution with $HAuCl_4$ followed by the addition of $AgNO_3$. The corresponding results are shown in Supporting Figure S10A–B and C–D. The cases differed by the reaction conditions; the concentration of chitosan solution was fixed at 2%, whereas the concentration of $HAuCl_4$ and $AgNO_3$ was 0.1% (Supporting Figure S10A–B) and 0.5% (in Supporting Figure S10C–D) per each reagent in these two cases and the reaction time was equal to 24 h and 48 h for first and second case, respectively. Note that the time to add $AgNO_3$ was also specific to these two cases. In order to determine the participation of Au and Ag in the formation of chitosan-Au-Ag hydrogels, ICP-MS analysis was performed for wet hydrogels that had previously been swollen to the maximum degree of swelling, thus releasing unbound Au and Ag. The remining solutions in the wells after chitosan, $HAuCl_4$ and $AgNO_3$ reactions were also analysed. The obtained results (Supporting Figure S10) indicate in both cases the dominant participation of Au in the formation of chitosan-Au-Ag hydrogels. It is also clear that not all reagents are consumed in the formation of hydrogels (Supporting Figure S10B and D). The higher the concentration of $HAuCl_4$ and $AgNO_3$, the more of these reagents remain in the wells after hydrogels are removed. It has also been observed that $AgNO_3$ remains in the wells in greater quantity than $HAuCl_4$. The wet hydrogels contained mainly Au rather than Ag (Supporting Figure S10A and C). It was calculated that 33.3% $HAuCl_4$ and 96.5% $AgNO_3$ is removed from the chitosan-Au-Ag hydrogel structure during the swelling process in the first reaction (Supporting Figure S10A and B). However, for the second analysed reaction (Supporting Figure S10C and D), the corresponding values are equal to 64.71 and 89.17%. This means that for higher reagents concentrations more

Au and Ag are bound, but also a significant amount is lost during hydrogel formation and swelling. This is a preliminary study, however, it points to possible further routes to obtain other types of chitosan hydrogels containing different metals as chitosan macromolecules crosslinkers.

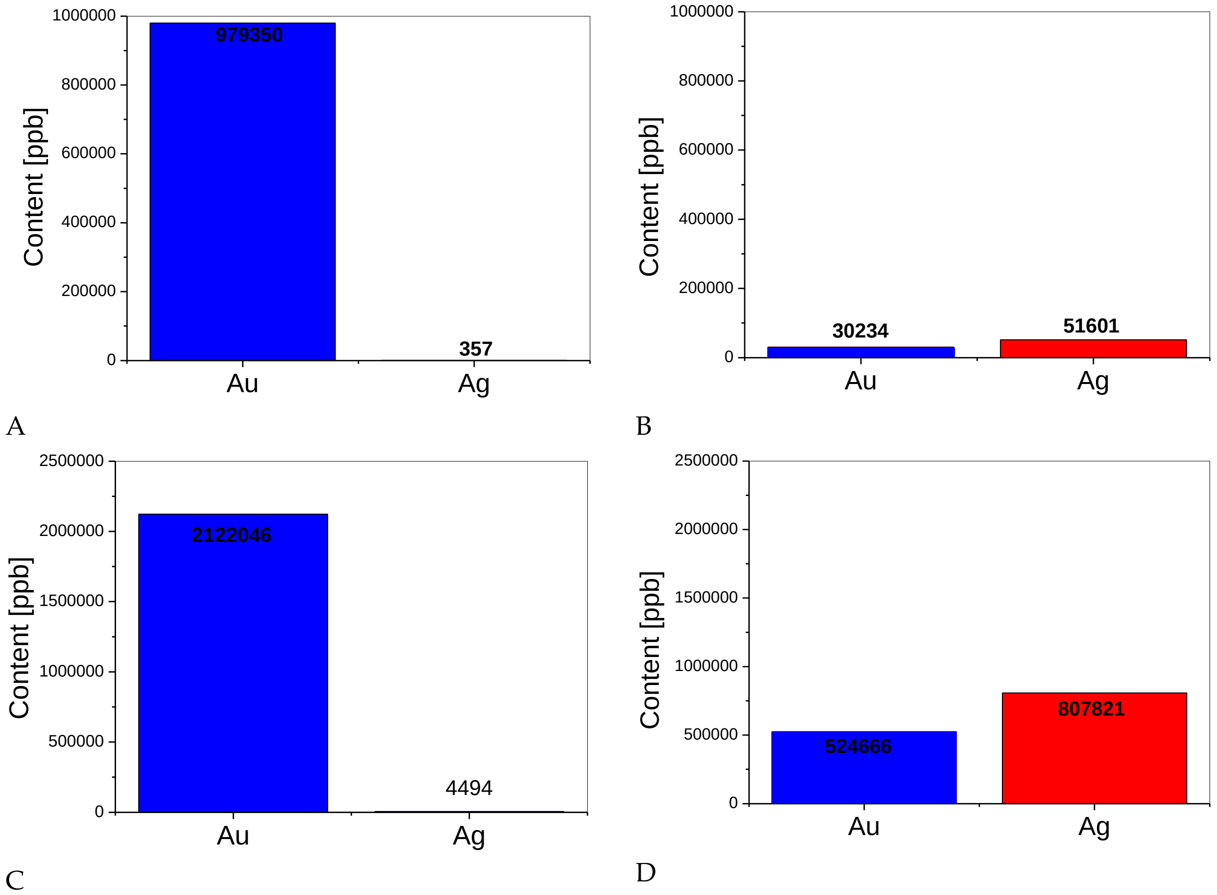

Supporting Figure S10. ICP-MS analysis of Au and Ag content for chitosan hydrogels formed during the reaction of 2% chitosan solution with 0.1% $AgNO_3$ and 0.1% $HAuCl_4$ (Fig. A and B; 24 hour reaction time; $AgNO_3$ added after 3 h of reaction between chitosan and $HAuCl_4$) and 0.5% $AgNO_3$ and 0.5% $HAuCl_4$ (C and D; 48 h reaction time; $AgNO_3$ added after 24 h of reaction between chitosan and $HAuCl_4$). A and C are the metal content in wet hydrogels, whereas B and D are the metal content of remaining solutions in the reaction wells the hydrogels are removed from the wells.

References

1. Kozicki, M.; Kołodziejczyk, M.; Szynkowska, M.; Pawlaczyk, A.; Lesniewska, E.; Matusiak, A.; Adamus, A.; Karolczak, A. Hydrogels made from chitosan and silver nitrate. Carbohyd. Polym. **2016**, *140*, 74–87. https://doi.org/10.1016/j.carbpol.2015.12.017
